# Dihydroartemisinin induces ferroptosis of hepatocellular carcinoma via inhibiting ATF4‐xCT pathway

**DOI:** 10.1111/jcmm.18335

**Published:** 2024-04-23

**Authors:** Jie Ji, Ziqi Cheng, Jie Zhang, Jianye Wu, Xuanfu Xu, Chuanyong Guo, Jiao Feng

**Affiliations:** ^1^ Department of Gastroenterology Shanghai Tenth People's Hospital, Tongji University School of Medicine Shanghai China; ^2^ Department of Gastroenterology Putuo People's Hospital, Tongji University Shanghai China; ^3^ Department of Gastroenterology Shidong Hospital, University of Shanghai for Science and Technology Shanghai China

**Keywords:** activated transcription factor 4, dihydroartemisinin, ferroptosis, hepatocellular carcinoma, solute carrier family 7 member 11, sorafenib

## Abstract

Management of hepatocellular carcinoma (HCC) remains challenging due to population growth, frequent recurrence and drug resistance. Targeting of genes involved with the ferroptosis is a promising alternative treatment strategy for HCC. The present study aimed to investigate the effect of dihydroartemisinin (DHA) against HCC and explore the underlying mechanisms. The effects of DHA on induction of ferroptosis were investigated with the measurement of malondialdehyde concentrations, oxidised C11 BODIPY 581/591 staining, as well as subcutaneous xenograft experiments. Activated transcription factor 4 (ATF4) and solute carrier family 7 member 11 (SLC7A11 or xCT) were overexpressed with lentiviruses to verify the target of DHA. Here, we confirmed the anticancer effect of DHA in inducing ferroptosis is related to ATF4. High expression of ATF4 is related to worse clinicopathological prognosis of HCC. Mechanistically, DHA inhibited the expression of ATF4, thereby promoting lipid peroxidation and ferroptosis of HCC cells. Overexpression of ATF4 rescued DHA‐induced ferroptosis. Moreover, ATF4 could directly bound to the SLC7A11 promoter and increase its transcription. In addition, DHA enhances the chemosensitivity of sorafenib on HCC in vivo and in vitro. These findings confirm that DHA induces ferroptosis of HCC via inhibiting ATF4‐xCT pathway, thereby providing new drug options for the treatment of HCC.

## 1 INTRODUCTION

Primary liver cancer (LC) is a group of malignancies with different histological features and prognosis that contains intrahepatic cholangiocarcinoma, hepatocellular carcinoma (HCC), combined hepatocellular‐cholangiocarcinoma and paediatric hepatoblastoma. According to *Global Cancer Statistics 2020*, primary LC is among the top three causes of cancer‐related death in 46 countries worldwide with global age‐standardised incidence and mortality rates of 9.5 and 8.7 per 100,000 people primary in 2020.^1^ The global LC‐related mortality rate is predicted to increase by 56.4% in 2040 as compared to 2020, accounting for an estimated 1.3 million deaths, suggesting an imminent burden to healthcare resources.^2^

HCC is the most common in primary LC, with a prevalence of 75%–85%.^3^ Although the pathogenesis and aetiology of HCC have not yet been fully elucidated, both have been linked to environmental and genetic factors.^4^ The causative factors of HCC include virus infection, non‐alcoholic fatty liver disease (NAFLD), alcoholic liver disease (ALD) and exposure to aflatoxins. Recent evidence has revealed that targeting ferroptosis could be an effective therapy for HCC.^5^, ^6^, ^7^ Sorafenib (SORA) is currently the first choice for therapy of advanced HCC. Numerous studies have demonstrated that SORA has the ability to induce ferroptosis in various cancer cell lines in vitro. Furthermore, it has been observed that the development of resistance to SORA is closely associated with the ferroptosis.^8^

Ferroptosis is a newly discovered cell death pathway characterised by excessive accumulation of lipid peroxides and iron‐dependent ROS.^9^, ^10^ Liver is the primary storage and metabolic organ of iron and is crucial in the process of maintaining iron homeostasis. However, liver is also highly sensitive to iron toxicity and imbalance of iron metabolism is a vital causative factor in HCC.^11^, ^12^ As a genetic factor, the incidence of HCC is 8% to 10% in patients with hereditary hemochromatosis, which is characterised by dysregulation of iron metabolism.^13^ Iron overload, which is a common phenomenon in chronic liver disease, not only directly damages the liver but also accelerates progression of the original liver disease, disrupts the tumour microenvironment and promotes progression of liver fibrosis to cirrhosis and even HCC.^14^, ^15^, ^16^ Therefore, it is reasonable to infer that the imbalance of iron metabolism is an important link in the pathogenesis of LC, and targeting ferroptosis of hepatoma cells can be an effective treatment for HCC.

Artemisinin is a sesquiterpene lactone compound containing a peroxy group extracted from *Artemisia annua*. Artemisinin and the commonly used derivatives, artesunate and artesunate, have significantly contributed to global malaria control, resulting in a cure for more than 1 million malaria patients.^17^ In honour of this achievement, Tu Youyou, who first discovered artemisinin, was awarded the 2015 Nobel Prize in Medicine or Physiology. Dihydroartemisinin (DHA) is an artificial semi‐synthetic derivative and is the primary active metabolite to retain antimalarial effects.^18^ In recent years, increasing attention has focused on the anti‐tumour effect of DHA in addition to its antimalarial function. A number of literatures have illustrated that DHA is beneficial for different cancers, including LC,^19^ pancreatic cancer,^20^ oesophageal cancer,^21^ leukaemia^22^ and colorectal cancer.^23^ The anti‐cancer mechanism of DHA mainly involves regulation of the cell cycle, suppression of cancer cell proliferation, promotion of apoptosis and inhibition of cancer cell metastasis. In addition, DHA is reported to disrupt iron metabolism, leading to cellular lipid peroxidation, which results in ferroptosis of cancer cells.^19^, ^24^ However, relatively few studies have explored the effect of DHA on inducing ferroptosis of HCC cells.

Although current treatments, including surgical excision, chemoradiotherapy and liver transplantation, have benefited patients with HCC, mortality remains to rise due to high recurrence rates and population growth. Hence, clarification of the mechanisms associated with the occurrence of HCC is urgently needed to screen for new drug targets. Therefore, our study was dedicated to explore the effect of DHA on HCC and to revealed the potential mechanisms, particularly induction of ferroptosis.

## 2 MATERIALS AND METHODS

### 2.1 RNA‐sequencing and bioinformatics analysis

The date from the Cancer Genome Atlas (TCGA) database (https://portal.gdc.cancer.gov/) were retrieved for all mRNA expression and clinical features analysed in this study. The dataset for analysis included 374 HCC specimens and 50 matched adjacent tissues. The normalised data were analysed with R software (http://www.r‐project.org). *p* Value < 0.05 was considered statistically significant.

### 2.2 Molecular docking

AutoDock Vina was used to analyse the binding affinities and modes of interaction between DHA and ATF4. The molecular structure of DHA was retrieved from PubChem Compound (https://pubchem.ncbi.nlm.nih.gov/). The 3D coordinate of ATF4 (PDB ID, 6IRR) was downloaded from the PDB (http://www.rcsb.org/pdb/home/home.do). Results were displayed with PyMOL2.

### 2.3 Drugs and reagents

DHA (purity ≥98% CAS: 71939‐50‐9) was bought from Yuanye Bio‐Technology Co., Ltd. (Shanghai, China). Puromycin was purchased from Sigma‐Aldrich Corporation (St. Louis, MO, USA). Hoechst 33342 fluorescence staining kit were purchased from Dojindo Laboratories Co., Ltd. (Kumamoto, Japan). Belnacasan, the protease inhibitor Z‐VAD‐FMK (benzyloxycarbonyl‐ValAla‐Asp (OMe) fluoromethylketone), ferrostatin‐1, erastin, deferoxamine and necrostatin‐1 were acquired from MedChemExpress (Monmouth Junction, NJ, USA). An apoptosis detection kit was got from BD Biosciences (San Jose, CA, USA). Foetal bovine serum (FBS) was obtained from Gibco (Thermo Fisher Scientific, Waltham, MA, USA).

### 2.4 Cell culture

SMMC‐7721, SMMC‐7721SR (Sorafenib‐resistant), Bel‐7402, HCC‐LM3, Huh7, HepG2, LO2 and HEK‐293 T cells were obtained from the Chinese Academy of Sciences Committee Type Culture Collection cell bank (Shanghai, China). All cells were cultured in Dulbecco's modified Eagle's medium (DMEM) supplemented with 10% FBS, penicillin (100 U/mL) and streptomycin (100 g/mL) at 37°C under a humidified atmosphere of 5% CO_2_.

### 2.5 Animal studies and in vitro assays

BALB/C male nude mice of 5 weeks old were obtained from the SLAC Experimental Animal Laboratory in Shanghai (Shanghai, China) and housed in a standard animal laboratory with free food and water. Obviously, logarithmic HCC‐LM3 cells or SMMC‐7721SR cells were suspended with serum‐free DMEM (3 × 10^6^/mL) and injected into the upper abdomen of mice (200 μL each). The calculation formula of tumour volume is: volume (mm^3^) = 0.5 × (long axis) × (small axis)^2^. When the xenograft tumour volume reached 100 mm^3^, the animals were randomly divided into the following groups:

To analyse the DHA effect, 10 mice were randomly divided into two groups (*n* = 5): (1) Normal control group (NC) mice were treated with normal solvent only; (2) DHA group was given DHA 100 mg/kg intragastric administration, once a day, for 20 days.

To verify the target of DHA, another nine mice were used. They were divided into three groups (*n* = 3): (1) EV group; (2) ATF4‐OE group; (3) ATF4‐OE + DHA group, which were injected with stable transfected ATF4‐OE SMMC‐7721 cells in the upper flank region.

For the combination treatment with SORA, 16 mice were randomly divided into four groups (*n* = 4): (1) NC group; (2) for the SORA group, mice were treated with 10 mg/kg SORA by gavage once a day for 20 days; (3) DHA group; (4) for the DHA + SORA group, mice were treated with both 100 mg/kg DHA and 10 mg/kg SORA by gavage once a day for 20 days.

At the end of the experiment, mice were anaesthetised with 1.25% pentobarbital (40 mg/kg, intraperitoneally injected), tumours were resected and imaged and then soaked in 4% paraformaldehyde. At the same time, the heart, kidney and lung of NC group and DHA group were separated for toxicity analysis.

The protocols for the CCK8 assay, EdU assay, western blotting, qRT‐PCR analysis, flow cytometry, haematoxylin and eosin staining, immumohistochemical staining, transmission electron microscopy, colony formation assay, plasmid construction, lentivirus packaging, as well as cell transfection are included in Data S1 and Tables S1–S3.

### 2.6 Malondialdehyde (MDA) concentration and the glutathione (GSH)/glutathione disulfide (GSSG) ratio

The MDA concentration was detected by a MDA assay kit (Dojindo Laboratories Co., Ltd., Kumamoto, Japan). GSH and GSSG assay kits (Beyotime Biotechnology, Shanghai, China) were used to measure the GSH/GSSG ratio.

### 2.7 Cell death detection and Lipid peroxidation assays

Lipid peroxidation assays and cell death detection were analysed as previously described.^25^ In summary, treated cells were cultured with 5 μM BODIPY™ 581/591 (GlpBio Technology, Inc., Montclair, CA, USA) or PI (BD Biosciences) for 5 or 30 min. And then collection of cells by centrifugation for flow cytometry detection.

### 2.8 Iron assay

FerroOrange and Mito‐FerroGreen probes (Dojindo Laboratories Co., Ltd.) were applied for assessing intracellular and mitochondrial iron levels, respectively. Briefly, cells were cultured with 40 μM DHA for 24 h, then washed with Hanks' balanced salt solution three times prior to the addition of 1 μM FerroOrange or 5 μM Mito‐FerroGreen. 10 μM Hoechst 33342 was used for nuclei staining and 1 μM MitoBright Red for mitochondria staining. After 30 min, the stained cells were photographed under a confocal laser scanning microscope (Carl Zeiss AG, Jena, Germany).

### 2.9 Immunofluorescence (IF) assay

Cells were cultured in confocal dishes and maintained with 40 μM DHA for 24 h, then fixed with 4% paraformaldehyde for 15 min and permeabilized with 0.3% Triton X‐100 for 20 min. After blocking with goat serum for 1 h, cells were incubated with a primary antibody against activated transcription factor 4 (ATF4) (dilution, 1:1000; Abcam, Cambridge, MA, USA) at 4°C overnight. The next day, cells were incubated with secondary antibody (Alexa Fluor 647 AffiniPure) for 30 min. The nuclei were stained with DAPI (Sigma‐Aldrich Corporation). Finally, a confocal laser scanning microscope was applied to obtain images of immunofluorescence.

### 2.10 Dual‐luciferase reporter assays

Using the JASPAR database to predict the potential binding sites of ATF4 in the promoter region of solute carrier family 7 member 11 (SLC7A11) (from −1000 bp to +200 bp). The SLC7A11 promoter region and corresponding mutations were subcloned into the pGL4.10 luciferase reporter vector (Promega Corporation, Madison, WI, USA). Then, ATF4‐OE 293 T or ATF4‐EV 293 T cells at confluence of 70%–80% were transiently co‐transfected with the constructed vector or the *Renilla* pRLTK plasmid (Promega Corporation) using Lipo8000™ transfection reagent (Beyotime Biotechnology) in a 24‐well plate. At 48 h after transfection, luciferase activity was tested with the Luciferase Reporter Assay Kit (Beyotime Biotechnology). *Renilla* luciferase activity was used to normalise Firefly luciferase activity.

### 2.11 Statistical analysis

Each experiment was repeated at least three times. All quantitative data are expressed as the mean ± SD. The unpaired, two‐tailed Student's *t*‐test was used for comparisons between two groups and one‐way analysis of variance was followed by Tukey's post‐hoc test for comparisons of three or more categorical, independent groups. A probability (*p*) value of <0.05 was considered statistically significant.

## 3 RESULTS

### 3.1 DHA exerts anti‐HCC effects in vitro

**FIGURE 1:**
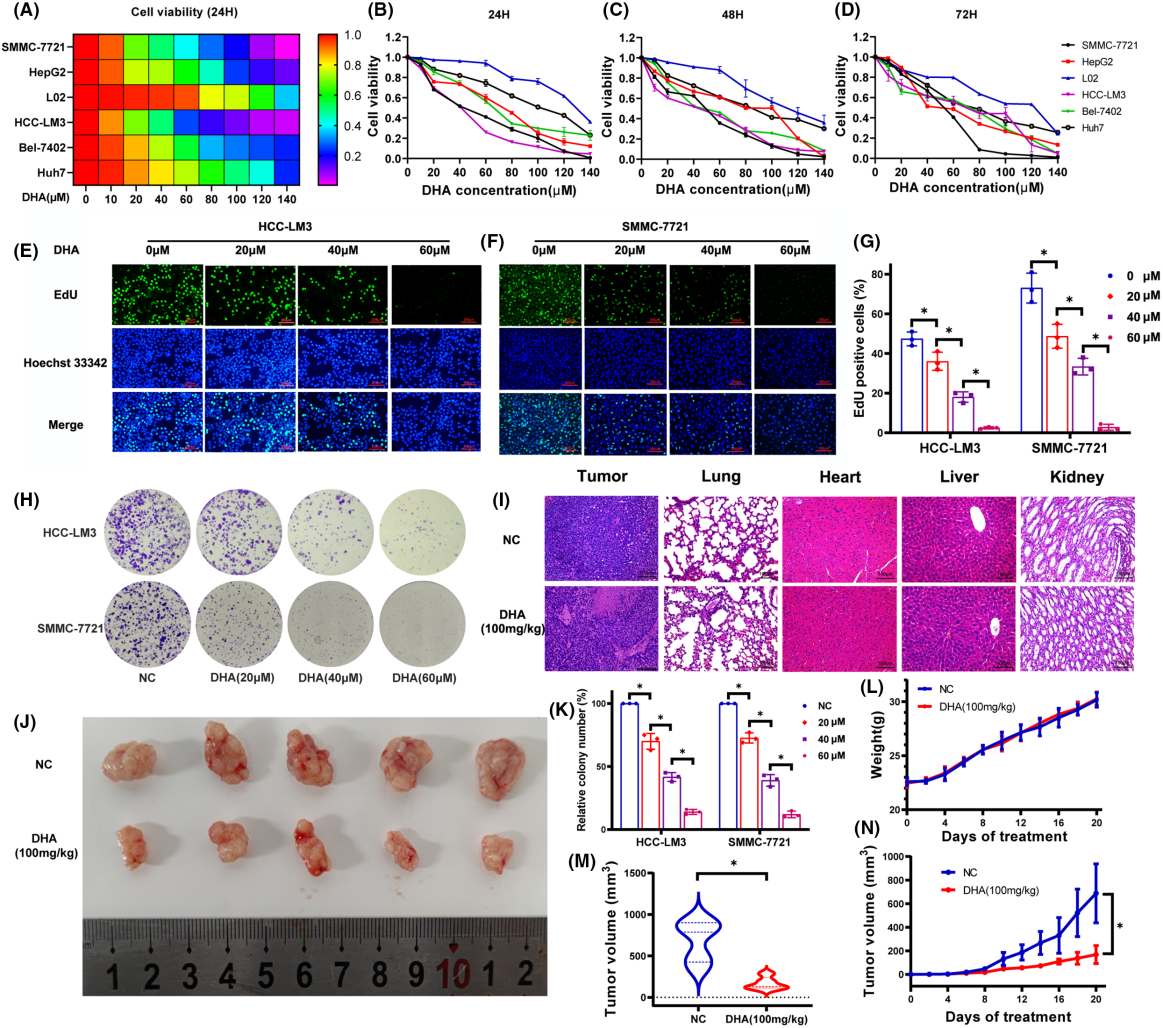
The effects of DHA on HCC in vitro and in vivo. (A) Indicated HCC cell lines were treated with vehicle or DHA for 24 h. Then the cell viability was determined by CCK‐8 assay. Cell viability data are presented as the means of 3 independent experiments in a heat map. (B–D) The cell viability was determined using the CCK8 assay after DHA treatment for 24, 48 and 72 h. (E, F) DNA synthesis in HCC‐LM3 and SMMC‐7721 treated with DHA for 24 h was evaluated by EdU incorporation. (G) The number of EdU‐positive cells in HCC‐LM3 and SMMC‐7721 was quantified. (H) Cell clone colonies formed by the HCC‐LM3 and SMMC‐7721 treated with DHA (0, 20, 40 and 60 μM). (I) The H&E staining of liver, tumour, heart, kidney and lung from the NC and DHA groups (original magnification, 200×). (J) The gross manifestation and volumes of tumours on day 20 after injection of HCC‐LM3 cells (*n* = 5). (K) The cell clone colonies formed by the HCC‐LM3 and SMMC‐7721 in each well were quantified. (L) Changes in mouse body weight were recorded at the indicated time points (*n* = 5, *p* > 0.05). (M) Differences in tumour volumes of nude mice on day 20 after injection of HCC‐LM3 cells. (N) Tumour volumes were recorded at the indicated time points. All quantitative data are shown as the mean ± SD from three independent experiments. *p < 0.05, **p < 0.01, ***p < 0.001.

The CCK8 assay was employed to assess the toxicity of DHA at different concentrations between normal liver cell line and HCC cells. DHA inhibited the viability of HCC cells in a time‐ and dose‐dependent manner (Figure 1A–D). DHA had rarely harmful effect on the viability of LO2 cells, indicating that DHA inhibited proliferation of HCC cells without injuring normal liver cells. SMMC‐7721 (IC_50_: 36.19 μM) and HCC‐LM3 (IC_50_: 41.38 μM) cells were sensitive to DHA at 24 h. Therefore, in the subsequent experiments, we chose HCC‐LM3 and SMMC‐7721 cells for in vitro study and 20, 40 and 60 μM DHA for the appropriate concentration. The colony formation and EdU assays confirmed that DHA inhibited the colony formation ability and proliferation of SMMC‐7721 and HCC‐LM3 cells (Figure 1E–H,K).

### 3.2 DHA inhibited the growth of xenograft tumour in vivo

A mouse xenograft model was established to explore whether DHA can also inhibit the growth of HCC cells in vivo. In Figure 1J,M,N, as compared to the normal control (NC) group, DHA administered at 100 mg/kg significantly reduced the tumour volume of mice but had no obvious effect on the weight of mouse (Figure 1L). Subsequent haematoxylin and eosin (HE) staining of the tumour tissues showed that NC group had a greater proportion of cancer cells in the tumour tissues, while the DHA group had more necrotic lesions (Figure 1I). These results suggest that DHA promoted necrosis of the xenograft tumour, thereby inhibiting growth and proliferation of LC cells. Further examinations showed that the concentrations of DHA used in the experiments had no toxic effects to the heart, liver, kidneys and lungs of mice (Figure 1I and Figure S1). Collectively, these results confirm the antitumor effect of DHA both in vitro and in vivo.

### 3.3 The anticancer mechanism of DHA involves induction of ferroptosis

**FIGURE 2:**
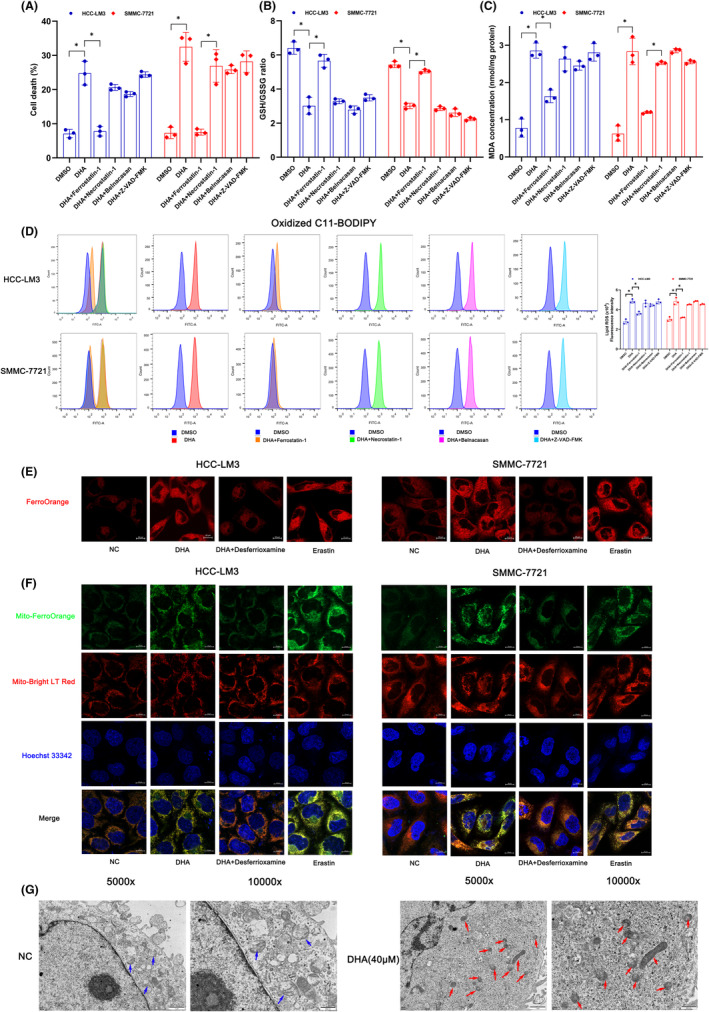
Inducing ferroptosis in HCC is the main anticancer way of DHA. (A) Cell death was determined via propidium iodide (PI) staining coupled with flow cytometry after treated with DMSO, DHA, DHA + ferrostatin‐1, DHA + Necrostatin‐1, DHA + Belnacasan or DHA + Z‐VAD‐FMK in HCC‐LM3 and SMMC‐7721. (B) The relative GSH/GSSG ratio was detected after treated with DMSO, DHA, DHA + ferrostatin‐1, DHA + Necrostatin‐1, DHA + Belnacasan or DHA + Z‐VAD‐FMK in HCC‐LM3 and SMMC‐7721. (C) MDA concentrations were determined after treated with DMSO, DHA, DHA + ferrostatin‐1, DHA + Necrostatin‐1, DHA + Belnacasan or DHA + Z‐VAD‐FMK in HCC‐LM3 and SMMC‐7721. (D) Lipid ROS levels were measured via BODIPY C11 staining coupled with flow cytometry. (E) Fe^2+^ in cytoplasm was measured via FerroOrange with confocal laser microscope images after treated with DHA, DHA + Deferoxamine (DFO) or Erastin. Scale bar = 10 μm. (F) Fe^2+^ in mitochondria was measured via Mito‐FerroGreen with confocal laser microscope images after treated with DHA, DHA + Deferoxamine (DFO) or Erastin. Scale bar = 10 μm. Erastin: 30 μM, Z‐VAD‐FMK: 10 μM, Necrostatin‐1: 1 μM, Belnacasan: 10 μM, Deferoxamine: 10 μM. (G) mitochondria under electron microscopy. Blue arrows indicate normal mitochondria. Red arrows indicate shrunken mitochondria with heavily condensed membrane. Scale bars, 500 nm. All quantitative data are shown as the mean ± SD from three independent experiments. *p < 0.05, **p < 0.01, ***p < 0.001.

To elucidate the main mechanisms employed by DHA to induce cell death in vitro, HCC cells were treated with common cell death mode‐specific inhibitors together with DHA. The results revealed that the proportion of dead cells and MDA expression were significantly increased (Figure 2A,C), while the GSH/GSSG ratio was significantly decreased (Figure 2B). Meanwhile, DHA inhibited the expression of glutathione peroxidase 4 (GPX4) and xCT (SLC7A11) (Figure S2), indicating that lipid peroxidation after treated with DHA could be closely related to ferroptosis. These changes were reversed by ferrostatin‐1, while the necroptosis inhibitor necrostatin‐1, pyrodeath inhibitor Belnacasan and apoptosis inhibitor Z‐VAD‐FMK failed to restore DHA‐induced lipid peroxidation (Figure 2A–C). These results reveal that the death of HCC cells induced by DHA occurs via ferroptosis.

Ferroptosis is characterised by excessive accumulation of iron‐dependent ROS and lipid peroxides. Therefore, iron levels in HCC cells and lipid peroxidation after DHA treatment were the key points of this study. C11 BODIPY 581/591 is a fluorescent probe used to test the level of lipid peroxidation. As is shown in Figure 2D, DHA treatment significantly increased the level of oxidised C11 BODIPY 581/591 as compared to solvent group, indicating that DHA indeed increased lipid peroxidation of HCC cells. Consistent with previous findings, only ferroptosis inhibitor prevented DHA‐induced oxidative stress. FerroOrange and Mito‐FerroGreen are a new class of fluorescent probes for detection of Fe^2+^ in cytoplasm and mitochondria, respectively. Confocal laser microscopy confirmed that FerroOrange intensity was obviously upregulated in DHA group compared to the control group, consistent with the erastin (ferroptosis inducer) treatment group. Meanwhile, the addition of deferoxamine reversed the trend of DHA‐induced accumulation of Fe^2+^ (Figure 2E). Staining with Mito‐FerroGreen demonstrated that DHA significantly increased the Fe^2+^ content in the mitochondria (Figure 2F). In addition, electron microscope analysis revealed that in the SMMC‐7721 cell line, tumour cells in the DHA‐treated group contained shrunken mitochondria with heavily condensed membrane, which are morphological features of ferroptosis (Figure 2G). Together, our study concurs that the anticancer mechanism of DHA in HCC cells involves induction of ferroptosis.

### 3.4 The induction of ferroptosis in HCC cells by DHA is associated with ATF4

**FIGURE 3:**
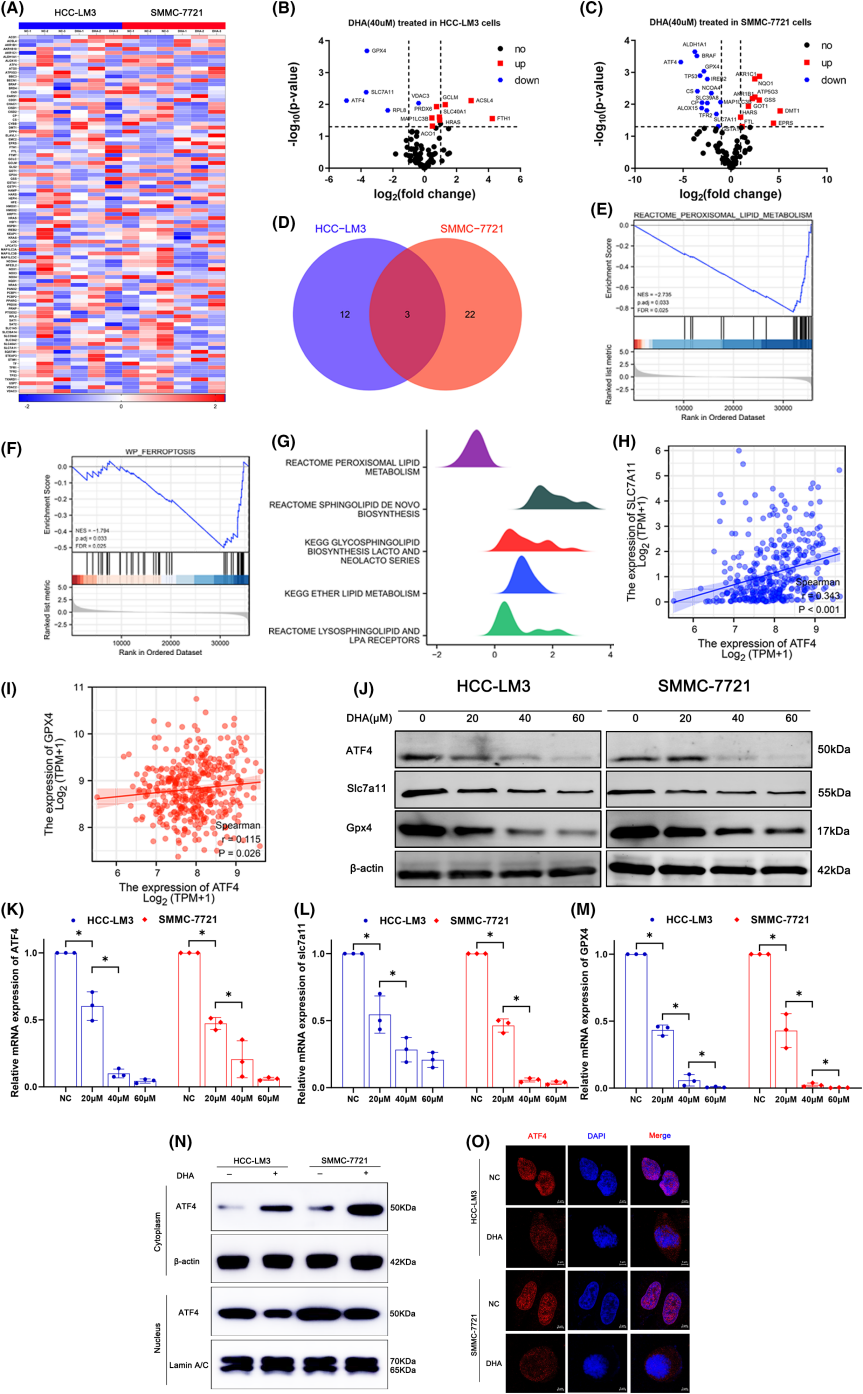
ATF4 is the main target gene of DHA‐induced ferroptosis in HCC. (A) Quantitative polymerase chain reaction array analysis of ferroptosis‐related genes in HCC‐LM3 and SMMC‐7721 after DHA treated (*n* = 3 samples per group). (B, C) The Volcano plot shows the differentially expressed genes of HCC‐LM3 and SMMC‐7721 treated with DHA. (D) Differentially expressed genes based on HCC‐LM3 and SMMC‐7721 are represented by a Venn diagram. (E, F) GSEA analysis in lipid peroxide metabolism and ferroptosis pathways. (G)The ridgeline plots showed that normal lipid metabolism was enhanced, but lipid peroxidation metabolism levels were decreased in the ATF4 high expression group. (H, I) Correlation analysis showed that ATF4 was positively correlated with SLC7A11 and GPX4. (J) Western blotting analysis of ATF4, SLC7A11 and GPX4 in HCC‐LM3 and SMMC‐7721 after 0, 20, 40 and 60 μM DHA treated. (K–M) Relative mRNA expression of ATF4, SLC7A11 and GPX4 in HCC‐LM3 and SMMC‐7721 after 0, 20, 40 and 60 μM DHA treated. (N) The cytoplasm and nuclear expression of ATF4 in HCC‐LM3 and SMMC‐7721 cells after 40 μM DHA treated. (O) The immunofluorescence staining of ATF4 in HCC cells. Scale bar = 5 μm. All quantitative data are shown as the mean ± SD from three independent experiments. *p < 0.05, **p < 0.01, ***p < 0.001.

To further seek the main mechanisms of DHA‐inducing ferroptosis in HCC cells, we performed PCR array to screen 90 ferroptosis‐related genes (Figure 3A and Tables S4 and S5). The results identified 15 differentially expressed genes in HCC‐LM3 cells (Figure 3B) and 25 differentially expressed genes in SMMC‐7721 cells (Figure 3C). A Venn diagram showed that the differentially expressed genes common to the two cell lines were ATF4, SLC7A11 and GPX4, with ATF4 as the most differentially expressed (Figure 3D). To evaluate the affinity of DHA to ATF4, we performed molecular docking analysis. Results showed that DHA could bound to ATF4 through visible hydrogen bonds and strong electrostatic interactions. For ATF4, DHA had low binding energy of −5.9 kcal/mol, indicating highly stable binding (Figure S3A,B).

Then, RNA sequencing data of 374 HCC patient specimens retrieved from TCGA were classified as high or low expression of ATF4. Gene set enrichment analysis demonstrated that the lipid peroxidation and ferroptosis pathways were mainly enriched in the low ATF4 expression group (Figure 3E,F). In addition, the ridgeline plots in Figure 3G show that normal lipid metabolism was enhanced, while lipid peroxidation was decreased in the high ATF4 expression group, further implicating ATF4 as an oncogene involved in the development of HCC.

Correlation analysis confirmed ATF4 was positively associated with SLC7A11 and GPX4 (Figure 3H,I), according to the qRT‐PCR results. Next, HCC cells were treated with 20, 40 and 60 μM DHA for qRT‐PCR and western blotting analyses. The results illustrated that DHA inhibited expression of ATF4 as well as xCT/SLC7A11 and GPX4 (Figure 3J–M), demonstrating an obvious positive correlation.

To identify the specific mechanism employed by DHA to down‐regulate ATF4 expression, cytoplasmic and nuclear proteins were extracted from HCC cells treated with DHA. The results indicated that the protein expression of ATF4 was decreased in the nucleus but upregulated in the cytoplasm of HCC cells (Figure 3N). Consistent with these results, IF staining showed greater fluorescence intensity of ATF4 in the nucleus than in the cytoplasm in NC group (Figure 3O). However, the fluorescence intensity of ATF4 was greatly rose in the cytoplasm but dramatically decreased in the nucleus after treating with DHA, suggesting that DHA promoted translocation of ATF4 to the cytoplasm. As a transcription factor, ATF4 mainly functions in the nucleus and can regulate downstream genes after translocation from the nucleus to the cytoplasm by DHA. The relatively increased ATF4 in the cytoplasm may then be degraded by the ubiquitin‐proteasome system. Collectively, above results confirmed that the induction of ferroptosis in HCC cells by DHA is associated with ATF4.

### 3.5 High ATF4 expression is related to poor clinicopathological prognosis of HCC

**FIGURE 4:**
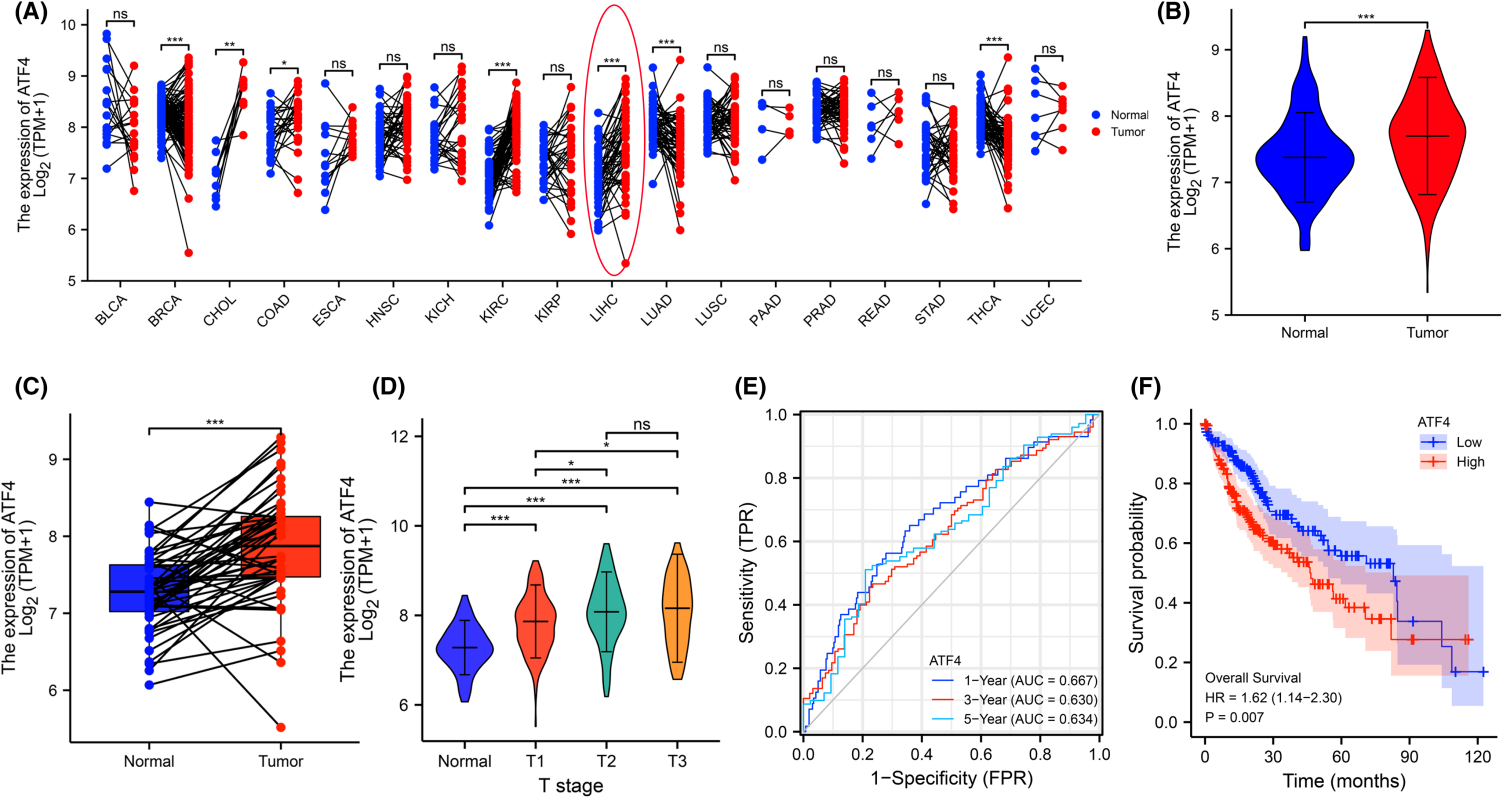
The high expression of ATF4 is associated with poor clinicopathological features of HCC. (A) Expression of ATF4 in 18 kinds of human cancers based on the TCGA database. (B) Expression of ATF4 in HCC tissues and adjacent normal tissues. (C) The matched analysis of ATF4 in HCC tissues. (D) Relationship between ATF4 expression and TNM‐stage in HCC. (E) Time‐ROC curve of ATF4 distinguishing HCC tissues. (F) Kaplan–Meier curves for overall survival. Data represent means ± SD, ns represents no statistical difference, *p < 0.05, **p < 0.01 and ***p < 0.001.

The expression patterns of ATF4 were investigated in 18 human cancers (Figure 4A). The results revealed that ATF4 was significantly lifted in liver hepatocellular carcinoma specimens, indicating that higher ATF4 was closely related to carcinogenesis. Assessment of the expression data of 374 HCC patient specimens and 50 paracancer tissues verified the higher expression of ATF4 in HCC tissues (Figure 4B), which was corroborated by matching analysis (Figure 4C). Moreover, analysis of the relationship of ATF4 with pathological stage, T, N, M stage, and histological grade revealed that increased ATF4 was related to poor clinicopathological features of HCC (Figure 4D, Table S6 and Figure S4A). Then, receiver operating characteristic (ROC) curve analysis confirmed ATF4 was a valid marker to differentiate HCC from normal patients ([AUC] = 0.783; Figure S4B), which was further verified by time ROC curve analysis (AUC > 0.63; Figure 4E). Kaplan–Meier analysis demonstrated that ATF4 was negatively related to disease‐specific survival (*p* = 0.01), progression‐free interval (*p* = 0.014) and overall survival (*p* = 0.007; Figure 4F and Figure S4C,D), further confirming the effective predictive performance of ATF4. Immunohistochemical results (Figure S5) also showed that HCC tissue had higher ATF4 expression than normal liver tissue. These results indicate that ATF4 is closely associated with clinicopathological prognosis of HCC with strong potential as a drug target.

### 3.6 Overexpression of ATF4 could rescue ferroptosis of HCC cells induced by DHA

**FIGURE 5:**
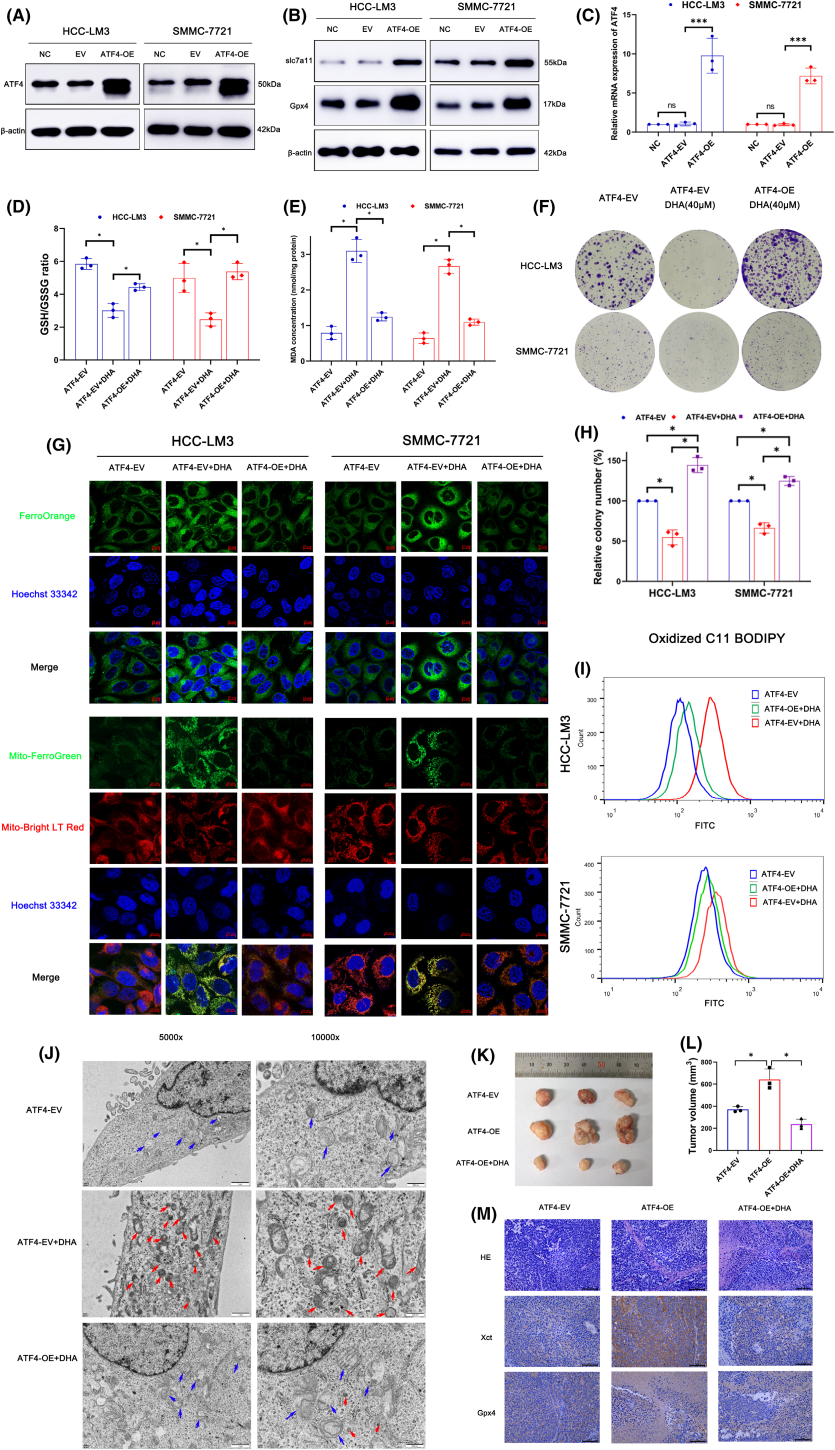
The overexpression of ATF4 could significantly rescue ferroptosis induced by DHA. (A) The transfection efficiency was verified by western blotting. (B) The expression of SLC7A11 and GPX4 in ATF4‐OE HCC cells by western blotting. (C) The transfection efficiency was verified by qRT‐PCR. (D) The relative GSH/GSSG ratio was detected in ATF4‐OE group with 40 μM DHA treated in HCC‐LM3 and SMMC‐7721 cells. (E) MDA concentrations were determined in ATF4‐OE group with 40 μM DHA treated in HCC‐LM3 and SMMC‐7721 cells. (F) Cell clone colonies formed in ATF4‐OE group with 40 μM DHA treated in HCC‐LM3 and SMMC‐7721 cells. (G) Fe^2+^ in cytoplasm and mitochondria were measured via FerroOrange and Mito‐FerroGreen with confocal laser microscope images. (H) The cell clone colonies formed by the HCC‐LM3 and SMMC‐7721 in each well were quantified. (I) Lipid ROS levels were measured via BODIPY C11 staining coupled with flow cytometry. (J) mitochondria under electron microscopy. Blue arrows indicate normal mitochondria. Red arrows indicate shrunken mitochondria with heavily condensed membrane. Scale bars, 500 nm. (K) The gross images of tumours in EV, ATF4‐OE and ATF4‐OE + DHA groups (L) The gross manifestation and volumes of tumours on day 20 after injection of SMMC‐721 cells (*n* = 3); (M) the H&E staining and IHC staining of xCT and GPX4. Scale bars: 100 μm. All quantitative data are shown as the mean ± SD from three independent experiments. *p < 0.05, **p < 0.01, ***p < 0.001.

To further verify that ATF4 is the target of DHA‐induced ferroptosis, lentiviruses including ATF4‐OE or an empty vector (EV) were transfected into HCC cells. Western blotting and qRT‐PCR assays were applied to confirmed transfection efficiency. ATF4 expression was obviously elevated in the ATF4 overexpression group, demonstrating excellent transfection efficiency (Figure 5A,C). In particular, protein levels of xCT/SLC7A11 and GPX4 were obviously elevated in the ATF4 overexpression group but not the EV group, suggesting that ATF4 may regulate expression of xCT/SLC7A11 and GPX4 (Figure 5B). The results of the colony formation assay confirmed that overexpression of ATF4 enhanced cell proliferation in the DHA + ATF4‐OE group compared to the DHA + ATF4‐EV group (Figure 5F,H). Furthermore, the effects of ATF4 overexpression on DHA‐induced ferroptosis were explored. The results confirmed that overexpression of ATF4 increased the GSH/GSSG ratio and reduced the MDA concentration (Figure 5D,E). Meanwhile, the cytoplasmic and mitochondrial contents of Fe^2+^ were obviously upregulated in the ATF4‐OE group, as determined with the FerroOrange and Mito‐FerroGreen probes (Figure 5G). The results of oxidised C11 BODIPY 581/591 showed that overexpression of ATF4 significantly reversed DHA‐induced lipid peroxidation (Figure 5I). In addition, transmission electron microscopy results also showed that overexpression of ATF4 could significantly save the DHA induced mitochondrial shrinkage and other ferroptosis manifestations (Figure 5J).

Furtherly, in vivo results shown that ATF4‐OE group displayed larger tumour volumes than the EV group, while DHA treatment could inhibit the tumour growth and proliferation effectively (Figure 5K,L). At the same time, H&E staining and IHC staining results showed that the expression levels of xCT and GPX4 were higher in the ATF4‐OE group (Figure 5M). This suggests that overexpression of ATF4 can promote the formation of tumour tissues in vivo by inhibiting the ferroptosis of tumour cells. Collectively, these results indicate that overexpression of ATF4 prevents ferroptosis by reducing intracellular iron content and inhibiting lipid peroxidation, and DHA effectively restrained the metastatic potential of HCC cells, demonstrating the antitumor effect of DHA in vitro and in vivo.

### 3.7 ATF4 regulates ferroptosis in HCC through xCT‐GSH‐GPX4 axis

**FIGURE 6:**
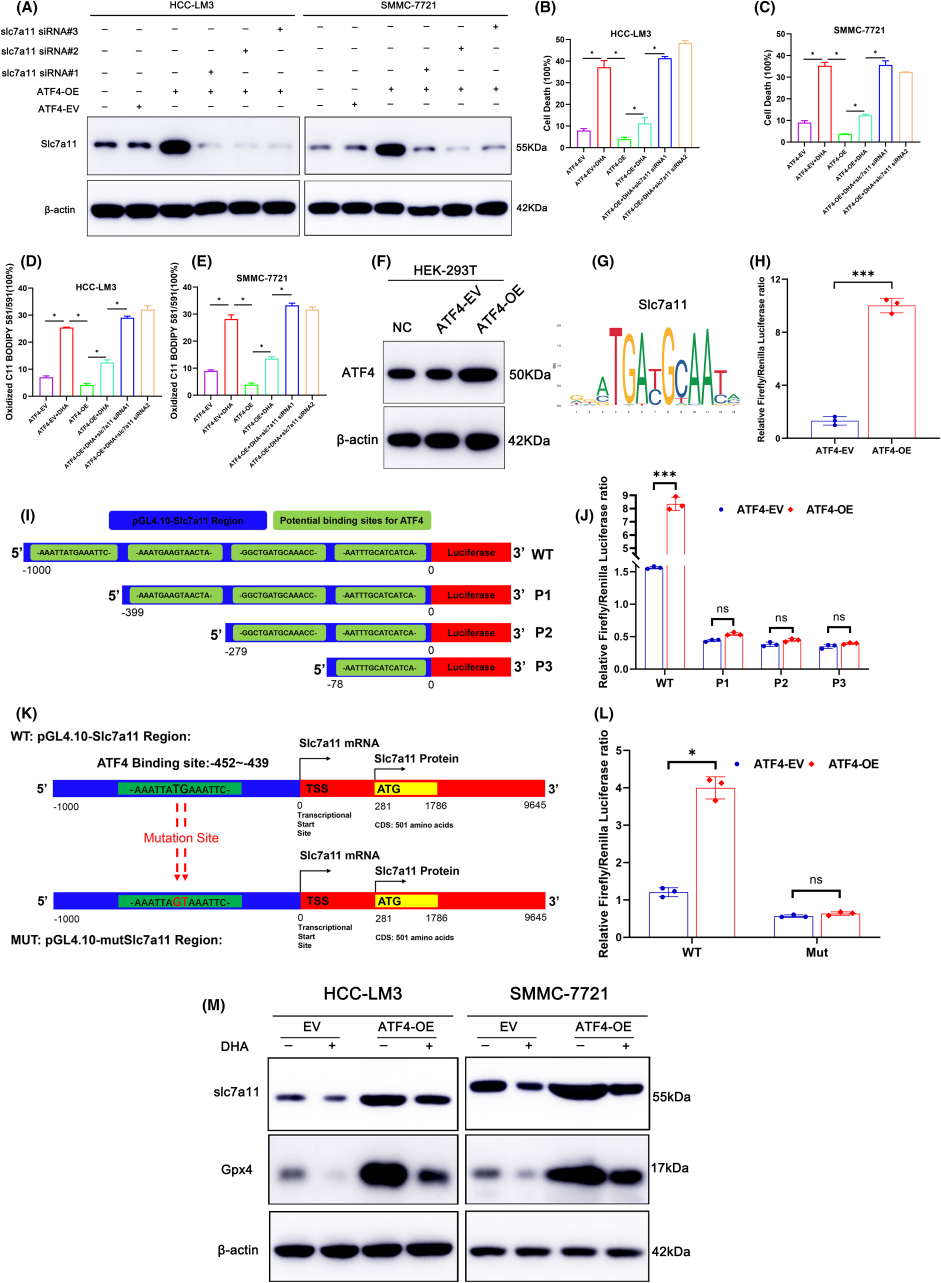
ATF4 regulates ferroptosis in HCC through xCT‐GSH‐GPX4 axis. (A) HCC‐LM3 and SMMC‐7721 cells were transfected with siRNAs targeting SLC7A11, and silencing efficiency was verified via western blotting. (B, C) Cell death was determined via propidium iodide (PI) staining coupled with flow cytometry in HCC‐LM3 and SMMC‐7721 cells. (D, E) Lipid ROS levels were measured via BODIPY C11 staining coupled with flow cytometry in HCC‐LM3 and SMMC‐7721 cells. (F) The transfection efficiency was verified by western blotting in HEK‐293 T. (G) The sequence logo of SLC7A11. (H) Relative luciferase activity in different groups determined by a dual‐luciferase reporter assay. *n* = 3 per group. (I) Schematic diagram of truncated mutants for possible binding sites in the SLC7A11 promoter region of ATF4. (J) Relative luciferase activity in different groups determined by a dual‐luciferase reporter assay. *n* = 3 per group. (K) Schematic diagram of possible binding sites in the SLC7A11 promoter region of ATF4 and mutation sites. (L) Relative luciferase activity in different groups determined by a dual‐luciferase reporter assay. *n* = 3 per group. (M) The expression of SLC7A11 and GPX4 in ATF4‐OE and ATF4‐EV HCC cells with 40 μM DHA treated. All quantitative data are shown as the mean ± SD from three independent experiments. *p < 0.05, **p < 0.01, ***p < 0.001.

Our previous study showed that overexpressed ATF4 significantly increased protein expression of xCT/SLC7A11, suggesting that ATF4 might directly regulate xCT/SLC7A11. For confirmation, HCC cells were transfected with siRNA targeting SLC7A11, which showed that silencing of SLC7A11 (Figure 6A) significantly increased the proportion of dead HCC cells and lipid peroxidation as compared to the ATF4‐OE + DHA group (Figure 6B–E), indicating that silencing of SLC7A11 reversed the rescue effect of ATF4 overexpression against DHA‐induced ferroptosis of HCC cells. To further verify that ATF4 targets SLC7A11, a dual‐luciferase reporter system was constructed. The JASPAR database predicted that the SLC7A11 promoter region contains multiple potential binding sites for ATF4 (Table S7). For confirmation, HEK‐293 T cells were transfected with lentiviruses for overexpression of ATF4 (Figure 6F), and the reporter plasmid pGL4.10‐SLC7A11 was constructed by cloning the 1000‐bp sequence upstream of the transcription start site of SLC7A11 into the plasmid pGL4.10. The results demonstrated that luciferase intensity was stronger in the ATF4‐OE group than the ATF4‐EV group, thereby confirming that ATF4 targets SLC7A11 (Figure 6G,H). Next, three truncated mutants of the 1000‐bp sequence upstream of the transcription start site of SLC7A11 were designed and cloned into the plasmid pGL4.10 based on the ranking score and locations of potential binding sites (Figure 6I). The results demonstrated that luciferase intensity in P1 group had sharply decreased, suggesting that the potential binding of ATF4 to the SLC7A11 promoter region was between −1000 and −399 bp (Figure 6J). Subsequently, the predicted binding site ‘AAATTATGAAATTC’ was mutated to ‘AAATTAGTAAATTC’, which is located between −1000 and −399 bp, and the mutant plasmid pGL4.10‐mut was constructed (Figure 6K). We could find that luciferase intensity was obviously upregulated in the mutant group, while there was no obvious change in the ATF4‐EV and ATF4‐OE groups (Figure 6L). Collectively, these results confirmed that ATF4 binds to the ‘AAATTATGAAATTC’ sequence in the SLC7A11 promoter region to promote transcription. The xCT‐GSH‐GPX4 axis plays an important role in cellular resistance to ferroptosis. In our study, DHA inhibits ATF4 expression, which further leads to inhibition of SLC7A11 transcriptional levels. At the same time, due to the decrease of glutamate/cystine transporter (xCT), the synthesis of intracellular GSH lacks cystine, which affects the expression of GPX4 to a certain extent and leads to the occurrence of ferroptosis. The western blotting results confirmed DHA down‐regulated the expression of xCT/SLC7A11 and GPX4. Meanwhile, DHA could reverse the increase of SLC7A11 and GPX4 caused by ATF4 overexpression (Figure 6M). Collectively, DHA induces ferroptosis of HCC via inhibiting ATF4‐xCT‐GSH‐GPX4 axis.

### 3.8 DHA enhanced the chemosensitivity of SORA on HCC in vivo and in vitro

**FIGURE 7:**
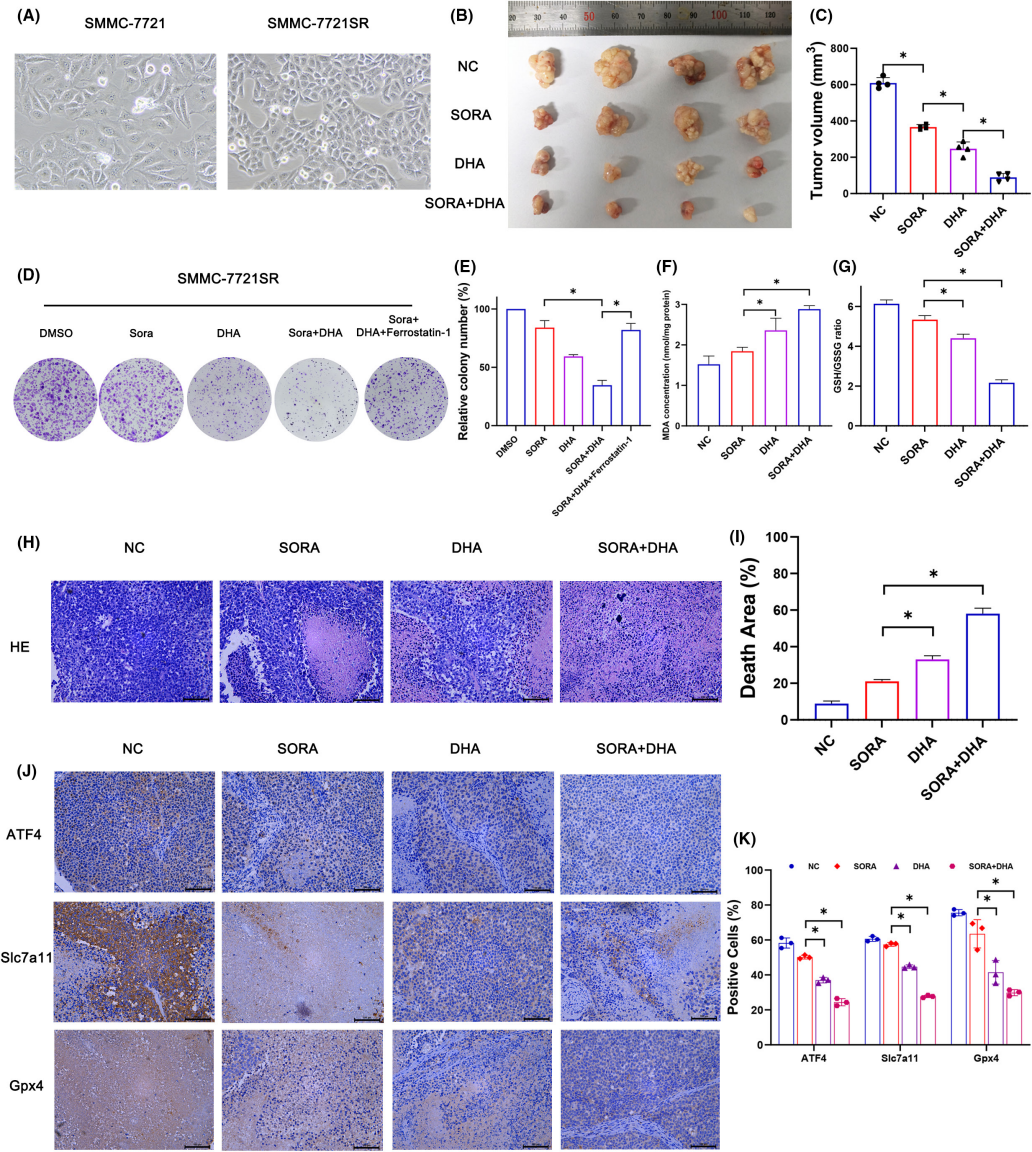
DHA enhanced the chemosensitivity of SORA on HCC in vivo and in vitro. (A) Morphology of SMMC‐7721 and SMMC‐7721SR at 200 × magnification. (B) The gross images of tumours in NC, SORA, DHA and SORA+DHA group. (C) The gross manifestation and volumes of tumours on day 20 after injection of SMMC‐721SR cells (*n* = 4). (D) Cell clone colonies formed in DMSO, SORA, DHA, SORA+DHA and SORA+DHA + ferrostatin‐1. (E) The cell clone colonies formed by SMMC‐7721SR in each well were quantified. (F) MDA concentrations were determined in NC, SORA, DHA and SORA+DHA group. (G) The relative GSH/GSSG ratio was detected in NC, SORA, DHA and SORA+DHA group. (H, I) The H&E staining and area of death in subcutaneous tumours in the four groups of mice. (J, K) Expression levels of ATF4, xCT and GPX4 in different treatment groups as determined by IHC analysis. All quantitative data are shown as the mean ± SD from three independent experiments. *p < 0.05, **p < 0.01, ***p < 0.001.

SORA resistance is a major obstacle for the treatment in patients with advanced HCC, and recent studies have reported that targeting ferroptosis can significantly reverse SORA resistance.^26^, ^27^, ^28^ Our previous study successfully constructed SMMC‐7721 Sorafenib‐resistant cell lines (SMMC‐7721SR).^26^ SMMC‐7721SR had a higher nucleo‐cytoplasmic ratio and higher malignancy than SMMC‐7721 (Figure 7A). Xenograft models showed that both SORA and DHA were effective in reducing xenograft tumour volume, but the combined treatment of DHA and SORA was more effective (Figure 7B,C). This suggests that DHA in combination with SORA can enhance the sensitivity of tumour cells to SORA. At the same time, in vitro colony formation assay showed that DHA can significantly inhibit the proliferation of SMMC‐7721SR cells, and the combination of DHA and SORA has a better effect. This inhibitory effect can also be blocked by ferroptosis inhibitor (Figure 7D,E). These suggest that DHA enhances HCC cells sensitivity to SORA by enhancing ferroptosis. We further examined the metabolic indexes related to ferroptosis and found that MDA levels were significantly increased in the combined treatment group, while GSH/GSSG was significantly decreased, which further confirmed the above conclusions (Figure 7F,G). In addition, H&E staining showed that there were more necrotic areas with large nucleus fragmentation in the DHA treatment group, while the above pathological changes were more obvious in the DHA and SORA combined treatment group (Figure 7H,I). Meanwhile, IHC staining results demonstrated that the expressions of ATF4, xCT and GPX4 in combination of SORA and DHA groups were obviously decreased (Figure 7J,K). Overall, these results suggested that DHA could increase SORA sensitivity to HCC cells by enhancing the ferroptosis pathway in vivo and in vitro.

## 4 DISCUSSION

The diagnosis and treatment of HCC are complicated by the multiple pathogenic factors and high heterogeneity.^29^ With the continued progress in molecular technologies and in‐depth research of the pathogenesis of HCC, a variety of new drugs targeting key genes have been developed to benefit HCC patients. However, due to population growth, frequent recurrence and drug resistance, management of HCC remains challenging.^3^ Ferroptosis is a newly discovered form of RCD that was first reported by Hirschhorn and Stockwell in 2012.^30^ Nowadays, targeting ferroptosis is becoming a viable choices for cancer therapy, particularly for malignant tumours that fail to respond to conventional treatments.^31^ Since dysregulation of iron metabolism is a crucial risk factor in HCC, more attentions have paid to the ferroptosis as a potential treatment strategy for HCC. A study by Liang et al. found that 26 genes related to ferroptosis were also indicators of the overall survival for HCC patients, suggesting that targeting ferroptosis‐related genes is a prospective therapeutic option for HCC.^29^ The present study confirmed the safety and efficacy of DHA against HCC and revealed that DHA induced ferroptosis by targeting the ATF4‐xCT pathway, thereby providing a promising drug choice for therapy of HCC.

DHA, known for its antimalarial effects, has received increasing concern as a potential anti‐tumour agent. A study by Bai et al.^23^ showed that DHA could restrain the proliferation of colorectal cancer cells by enhancing apoptosis and tumour cell cycle arrest. Moreover, DHA was confirmed to reverse osimertinib resistance in non‐small‐cell lung cancer by lifting the production of ROS and inhibition of heme.^32^ Notably, DHA is also confirmed to impair iron homeostasis and induce tumour cells ferroptosis. Du et al. verified that DHA integrated with cisplatin enhanced cisplatin sensitivity of pancreatic ductal adenocarcinoma, exhibiting an obvious synergistic effect related to ferroptosis.^33^ In our study, HCC cells treated with ferrostatin‐1, Z‐VAD‐FMK, necrostatin‐1 and Belnacasan revealed that DHA induced ferroptosis in HCC cells in vitro. At the same time, a mouse xenograft model verified that DHA inhibited tumour growth with no distinct toxicity to the main organs in vivo.

Ferroptosis is the main way of DHA causing the death of HCC cells, but its main mechanism and targets are rarely studied. In order to find the main target gene of DHA, PCR array assay containing 90 ferroptosis‐related genes was performed in SMMC‐7721 and HCC‐LM3 cells after DHA treatment. In our study, ATF4 was identified as the most differentially expressed gene. As a stress‐induced transcription factor, ATF4 is usually upregulated in a variety of diseases. ATF4 regulates the transcriptions including protein homeostasis, resistance to oxidative stress, and amino acid metabolism, allowing cells to tolerate stressors, such as hypoxia and amino acid restriction.^34^ In fact, ATF4 is often utilised in tumour cells to reduce stress caused by rapid proliferation and nutrient deficiency.^35^ In addition, ATF4 and related target genes are associated with angiogenesis and metastasis.^36^, ^37^ The results of the present study using TCGA data established that increased expression of ATF4 is related to worse clinicopathological features of HCC. Recent researches have verified the effects of ATF4 in ferroptosis of cancer cells. A study by Chen et al. illustrated that ATF4 prompted tumour proliferation, angiogenesis and malignancy of primary brain tumours via xCT/SLC7A11, a crucial regulator gene of the ferroptosis.^38^ In the present study, DHA reduced expression of ATF4 by inducing degradation and cytoplasm translocation, thereby inhibiting growth of HCC cells in vitro. Overexpression of ATF4 prevented ferroptosis by inhibiting lipid peroxidation and reducing intracellular iron, demonstrating that DHA can effectively reduce the metastatic potential of HCC.

SLC7A11, also called as System Xc^−^ or xCT, and GPX4 are important regulators of ferroptosis. The major effects of xCT are that it transfers glutamate from within the cell to the extracellular space and cystine from the extracellular space to within the cell.^39^ Intracellular cystine is used for the synthesis of GSH. Inhibition of xCT expression by an inducer of ferroptosis, such as erastin, decreases the synthesis of GSH, leading to the inability of GPX4 to reduce lipid peroxidation, resulting to ferroptosis.^40^ GPX4 is the only GPX member equipped to convert phospholipid hydroperoxides to phospholipid alcohols.^31^ Drug inhibition or genetic deletion of GPX4 can induce lipid peroxidation and subsequent ferroptosis.^41^ The xCT‐GSH‐GPX4 axis is considered the primary pathway to resist ferroptosis.^31^ An investigation of the regulatory effects of ATF4 found that DHA treatment not only down‐regulated expression of ATF4 but also LC7A11 and GPX4. In fact, ATF4 was positively correlated with SLC7A11 and GPX4 (Figure 3H,I), consistent with the qRT‐PCR results. Subsequently, it was surprising that the expressions levels of SLC7A11 and GPX4 were also up‐regulated by overexpression of ATF4, while silencing of SLC7A11 reversed the protective effect of overexpression of ATF4, leading to severe ferroptosis. Finally, the double luciferase assay confirmed that ATF4 is directly bound to the ‘AAATTATGAAATTC’ sequence of the SLC7A11 promoter region to promote transcription. Collectively, the present study confirmed DHA induces ferroptosis in HCC via inhibiting ATF4‐xCT‐GPX4 axis.

As a first‐line drug for advanced HCC, SORA acts on multiple targets and can significantly prolong the survival time of patients with advanced HCC. However, acquired resistance to sorafenib limits its clinical efficacy.^42^, ^43^ GAO et al. demonstrated that YAP/TAZ maintained the protein stability, nuclear localization and transcriptional activity of ATF4, which acted as key drivers of SORA resistance in HCC.^27^ In fact, restoring the sensitivity of HCCs to ferroptosis may be an important strategy for tackling SORA resistance. Sun et al. found that metallothionein (MT)‐1G is a key regulator of sorafenib resistance in human HCC cells. Knocking down MT‐1G increases glutathione depletion and lipid peroxidation, leading to sorafenib‐induced ferroptosis.^28^ In our study, we demonstrated that DHA can restore HCCs sensitivity to SORA by enhancing the ferroptosis pathway in vivo and in vitro. Accordingly, DHA may act as a promising chemotherapy agent combining with SORA for the treatment of HCC.

## 5 CONCLUSION

**FIGURE 8:**
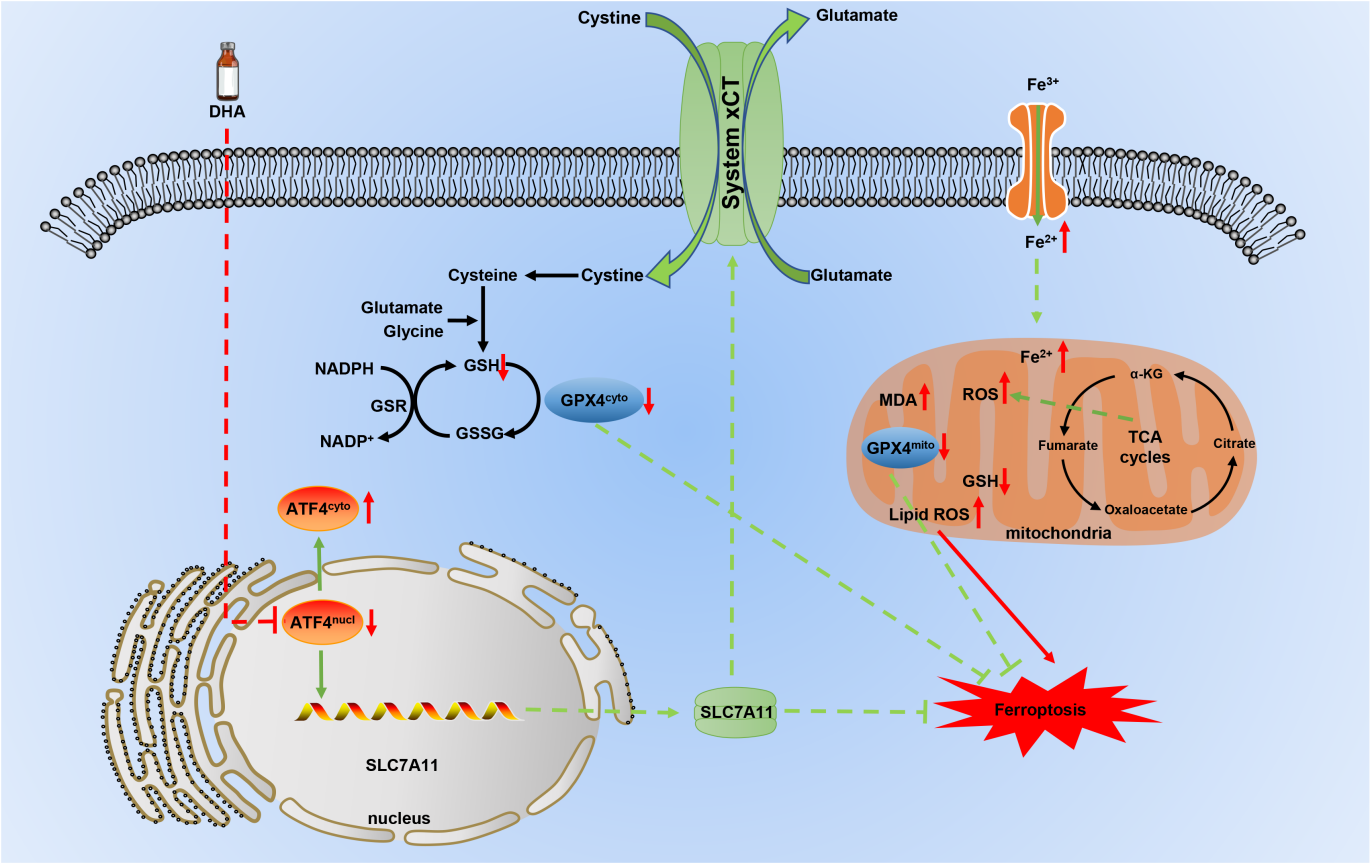
Schematic diagram of DHA targeting ATF4 induces xCT‐regulated ferroptosis in hepatocellular carcinoma.

This study provides new drug options for HCC therapy. Mechanistically, DHA inhibited expression and promoted cytoplasmic translocation of ATF4 to promote lipid peroxidation and induce ferroptosis in HCC cells. Overexpression of ATF4 rescued DHA‐induced ferroptosis. In addition, DHA enhances the chemosensitivity of SORA on HCC. Overall, DHA induces ferroptosis of HCC via inhibiting ATF4‐xCT pathway and act as a promising chemotherapy agent combining with SORA. (Figure 8).

## AUTHOR CONTRIBUTIONS

**Jie Ji:** Conceptualization (equal); investigation (equal); methodology (equal); writing – original draft (equal). **Ziqi Cheng:** Investigation (equal); methodology (equal); visualization (equal). **Jie Zhang:** Investigation (equal); methodology (equal); visualization (equal). **Jianye Wu:** Conceptualization (equal); investigation (equal); writing – original draft (equal). **Xuanfu Xu:** Supervision (equal); writing – review and editing (equal). **Chuanyong Guo:** Conceptualization (equal). **Jiao Feng:** Supervision (equal); writing – review and editing (equal).

## CONFLICT OF INTEREST STATEMENT

The authors declare that they have no competing interests.

## Supporting information


Data S1.

Tables S1–S7.

Figures S1–S5.

## Data Availability

All the data obtained and/or analysed during the current study were available from the corresponding authors on reasonable request.
