# Supplementary material for: Dihydroartemisinin induces ferroptosis of hepatocellular carcinoma via inhibiting ATF4‐xCT pathway
Source: J Cell Mol Med. 2024 Apr 23;28(8):e18335. doi: 10.1111/jcmm.18335 (PMC11037408; doi:10.1111/jcmm.18335)
Supplement: Supplementary file 1 — Data S1. [file JCMM-28-e18335-s001.pdf]

Supplementary methods

CCK8 assay

The cells in logarithmic phase were seeded in 96-well plates at a density of 2 × 104/mL for 24 h. Changing the culture medium and then DHA was added at concentrations of 0, 10, 20, 40, 60, 80, 100, 120, or 140 μM for 24, 48, or 72 h. Cell viability was then measured using the CCK8 assay according to the manufacturer's protocol. The half-maximum inhibition concentration (IC50) of DHA were calculated using the CalsuSyn software.

Colony formation

Cells were seeded at 1000 cells/well in 6-well plates for 7 days, and then 20, 40, and 60 μM DHA was added to treat the cells for another 7 days. When colony formation was evident, the cells were cleaned three times with PBS and then fixed with paraformaldehyde for 15 minutes and stained with 0.1% Crystal Violet for 10 minutes.

EdU assay

EdU assay was performed using an EdU kit (BeyoClick™ EdU Cell Proliferation Kit with Alexa Fluor 488, Beyotime, China). Briefly, SMMC-7721 and HCC-LM3 cells were seeded in 24-well plates and cultured in 20, 40, and 60 μM DHA for 24h. Subsequently, cells were incubated with EdU for 3 h, fixed with 4% paraformaldehyde for 15 min, and permeated with 0.3% Triton X-100 for another 15 min. The cells were incubated with the Click Reaction Mixture for 30 min at room temperature in a dark place and then incubated with Hoechst 33342 for 10 min.

Flow cytometry analysis of apoptosis

The cells were inoculated into 6-well plates for 24 h and treated with 40 uM DHA for 24 h. Cells were harvested, stained with annexin V-FITC and PI, and detected by flow cytometry. The results are analyzed by FlowJo software (version 10; FlowJo, Ashland, OR, USA). Annexin V positive and PI negative cells are considered early apoptotic cells and annexin V positive and PI positive cells are considered late apoptotic/secondary necrotic cells.

Western blotting

Total proteins were isolated from treated cells using RIPA Lysis buffer (Beyotime) supplemented with phosphatase and protease inhibitors on ice for 30 min. The nuclear and cytoplasmic protein was extracted using a commercial kit (Nuclear and Cytoplasmic Protein Extraction Kit, Yeasen Biotechnology, Shanghai, China). The protein concentration was quantified using a BCA protein assay kit (Yeasen Biotechnology, Shanghai, China). After boiled in 6×loading buffer (Beyotime), the proteins were separated by SDS-PAGE and transferred onto a nitrocellulose membrane (GE Healthcare). The membrane was blocked with 5% nonfat milk (Beyotime) in PBST for 1 h, and was then incubated with primary antibodies (Additional file 3: Table S1) overnight at 4°C. The membrane was washed with PBST for 3 times prior to incubation with goat anti-rabbit or goat anti-mouse secondary antibodies at room temperature. Finally, the chemiluminescent signal was detected via an ECL method or Odyssey (Licor, Lincoln, NE, USA).

Quantitative real-time PCR (RT-qPCR) assays and qPCR arrays

Total RNA was extracted from treated cells using the TRIzol reagent (Invitrogen) according to manufacturer's instructions. Reverse transcription was performed to synthesize cDNA using the PrimeScriptTM RT reagent Kit (TaKaRa). RT-qPCR was performed using KAPA SYBR® FAST qPCR Kit Master Mix (2X) Universal (Applied Biosystems) on the ABI 7500 real-time PCR System (Applied Biosystems). β-actin were used to normalize the expression levels of target genes. The 2-ΔΔCt method was employed to calculate the relative expression of target genes. The primers used are listed in Additional file 3: Table S1.

Quantitative PCR arrays are designed to analyse a panel of ferroptosis-related genes in DHA treated cells following the instructions of the manufacturer (Wcgene Biotechnology Corporation, China). Genes undetectable for three times were excluded.

Plasmid construction, lentivirus packaging, siRNAs construction and infection

The ATF4-overexpressing lentivirus was synthesized by Genechem (Shanghai, China). A full-length cDNA encoding the ATF4 sequence was amplified from 293T cDNA and then cloned into the CMV enhancer-MCS-sv40-puromycin vector. Empty vector (EV) was employed as the negative control. All recombinant lentiviruses were then generated from HEK-293 T cells using calcium phosphate precipitation. To establish a stable lentivirus transfection cell line, HCC-LM3,293T and SMMC-7721 cells were seeded in 6-well plates, and when 60–70% confluent, they were transfected with EV and ATF4 lentivirus for 12 h. The positive cells were selected by puromycin and transfection efficiency was determined by qPCR and western blotting.

Slc7a11 specific siRNA-1, 2 and 3 were synthesized by Tsingke Biotechnology Co., Ltd. (Beijing, China). The target sequences used in this study are shown as follows: slc7a11-siRNA-1, sense 5'-GGAGUUAUGCAGCUAAUUA-3' and antisense 5'-UAAUUAGCUGCAUAACUCC-3'; slc7a11-siRNA-2, sense 5'-CUACUUUACGACCAUUAAU-3' and antisense 5'-AUUAAUGGUCGUAAAGUAG-3'; slc7a11-siRNA-3, sense 5'-GAAUCUUCAUCUCUCCUAA-3' and antisense 5'-UUAGGAGAGAUGAAGAUUC-3'.

The siRNAs were transfected into HCC cells using Lipofectamine 8000 (Beyotime) in accordance with the manufacturer's protocol. The transfection efficiency was verified by Western blotting.

Hematoxylin and eosin (H&E) staining

The tissues soaked in paraformaldehyde were embedded in paraffin wax and cut into 3μm thick slices. During H&E staining, sections were stained with hematoxylin for 10 min and eosin for 5 min to observe the tissue damage.

Immumohistochemical (IHC) staining

Tumor sections (3 μm thick) were dewaxed and dehydrated. After antigen retrieval process and blocking, the sections were incubated with primary antibodies against ATF4, Xct and Gpx4. The slices were then incubated with suitable secondary antibodies. The positive staining area was observed under light microscope.

Transmission electron microscopy (TEM)

Samples were fixed with a solution containing 3% glutaraldehyde and 2% paraformaldehyde in 0.1M cacodylate buffer (pH 7.3), then washed in 0.1M sodium cacodylate buffer, treated with 0.1% Millipore-filtered cacodylate buffered tannic acid, postfixed with 1% buffered osmium, and stained en bloc with 1% Millipore-filtered uranyl acetate. The samples were dehydrated in increasing concentrations of ethanol, infiltrated, and embedded in LX-112 medium. The samples were polymerized in a 60 °C oven for approximately 3 days. Ultrathin sections were cut using a Leica Ultracut microtome (Leica, Deerfield, IL), stained with uranyl acetate and lead citrate in a Leica EM Stainer, and examined using a JEM 1010 transmission electron microscope (JEOL, USA, Inc., Peabody, MA) at an accelerating voltage of 80 kV. Digital images were obtained using the AMT Imaging System (Advanced Microscopy Techniques Corp, Danvers, MA) at MD Anderson's High-Resolution Electron Microscopy Facility.
