# Supplementary material for: Dihydroartemisinin induces ferroptosis of hepatocellular carcinoma via inhibiting ATF4‐xCT pathway
Source: J Cell Mol Med. 2024 Apr 23;28(8):e18335. doi: 10.1111/jcmm.18335 (PMC11037408; doi:10.1111/jcmm.18335)
Supplement: Supplementary file 2 — Tables S1–S7. [file JCMM-28-e18335-s002.pdf]

Table S1

The antibody used in this study.

antibody	Species	Targeted species	Supplier	Catalogue number
β-actin	Rbt	H, M, R	Abclonal	AC026
xCT	Rbt	H, M, R	Abcam	ab175186
ATF4	Rbt	H	Abcam	ab184909
GPX4	Rbt	H, M, R	Abmart	T56959
Lamin A/C	Rbt	H, M, R	PT	10298-1-AP

Abbreviations: H, human; M, mouse; Rbt, rabbit; R, rat; PT, Proteintech (Chicago, IL, USA).

Table S2

The primers for qRT-PCR in this study.

**Gene symbol**	**Forward primer**	**Reverse primer**
β-actin	GTGACGTTGACATCCGTAAAGA	GCCGGACTCATCGTACTCC
xCT	TCTCCAAAGGAGGTTACCTGC	AGACTCCCCTCAGTAAAGTGAC
ATF4	CCCTTCACCTTCTTACAACCTC	TGCCCAGCTCTAAACTAAAGGA
GPX4	GAGGCAAGACCGAAGTAAACTAC	CCGAACTGGTTACACGGGAA

Table S3

siRNA sequences

**Gene symbol**	**Target sequence**
SLC7A11-siRNA1	GGAGUUAUGCAGCUAAUUA
SLC7A11-siRNA2	CUACUUUACGACCAUUAAU
SLC7A11-siRNA3	GAAUCUUCAUCUCUCCUAA

Table S4.

The results of PCR array in HCC-LM3

Target Name	HCC-LM3-DHA/HCC-LM3-NC	P value	
ACO1	1.924513911	0.031114862	*
ACSL4	7.500789339	0.007544576	**
AKR1B1	1.455590021	0.370827388	ns
AKR1B10	0.841514242	0.827912729	ns
AKR1C1	1.205962225	0.652843736	ns
ALDH1A1	1.061998566	0.893144805	ns
ALOX15	0.994277008	0.990216558	ns
ATF4	0.033423195	0.007546188	**
ATG5	1.500340476	0.210733573	ns
ATP5G3	0.700416803	0.455915569	ns
BBC3	0.600497358	0.249711889	ns
BECN1	0.85184599	0.762130978	ns
BRAF	1.057623753	0.877740625	ns
BRD4	1.88795412	0.11416396	ns
CA9	1.002748096	0.995004805	ns
CARS1	0.533923025	0.583688444	ns
CDO1	0.53742211	0.563516226	ns
CHAC1	1.036358849	0.918951369	ns
CISD1	0.556410317	0.245623865	ns
CISD2	1.512700996	0.358792037	ns
CP	0.712693697	0.073651767	ns
CS	1.168847569	0.773210741	ns
CYBB	2.722700073	0.355350661	ns
DMT1	2.285070641	0.267934679	ns
DPP4	0.503167963	0.402210085	ns
ELAVL1	0.968163339	0.944072953	ns
EMC2	1.987702463	0.04034244	*
EPRS	0.773109749	0.691478029	ns
FTH1	18.85047705	0.027620354	*
FTL	1.382932822	0.378878117	ns
FTMT	0.780243831	0.451046177	ns
GCLC	2.120389837	0.586488504	ns
GCLM	2.444939225	0.010134913	*
GLS2	0.66393205	0.331374365	ns
GOT1	1.417244798	0.653928863	ns
GPX4	0.08109934	0.000208458	***
GSS	1.173089344	0.828648728	ns
GSTA1	0.689324701	0.240705891	ns
GSTP1	1.450091564	0.628757209	ns
HAMP	0.661239048	0.615635476	ns

HARS	0.721053277	0.642096405	ns
HEPH	0.776444006	0.511618	ns
HFE	0.45166601	0.134307343	ns
HMOX1	0.675823382	0.509914297	ns
HMOX2	0.962718723	0.971878965	ns
HRPT1	0.809154662	0.508501758	ns
HRAS	1.40086206	0.047565209	*
HSF1	0.799502463	0.429074635	ns
HSPB1	0.528458606	0.427254788	ns
IREB2	0.954557332	0.859536011	ns
KEAP1	0.853683354	0.199136834	ns
KRAS	1.022419001	0.917357136	ns
LOX	1.573460382	0.34203305	ns
LPCAT3	0.447769175	0.379745897	ns
MAP1LC3A	0.467423882	0.219406804	ns
MAP1LC3B	1.367900845	0.026422787	*
MAP1LC3C	0.888447972	0.245842196	ns
NCOA4	0.573651309	0.073306677	ns
NFE2L2	0.701157368	0.099021844	ns
NOX1	0.282476561	0.356297333	ns
NOX3	0.950020488	0.819695022	ns
NOX4	2.929226964	0.335388956	ns
NQO1	1.078393975	0.796169964	ns
NRAS	0.831735999	0.342302331	ns
PANX2	1.628330027	0.055209748	ns
PCBP1	0.921894916	0.814387242	ns
PCBP2	1.036269525	0.907799852	ns
PPARG	0.69397865	0.395855662	ns
PRDX6	1.67570561	0.011667114	*
PRNP	1.027674765	0.958056222	ns
PTGES2	0.83304717	0.49788674	ns
RPL8	0.201187326	0.015275015	*
SAT1	1.272668588	0.371421115	ns
SAT2	0.579281292	0.069758953	ns
SLC1A5	1.05027961	0.840815911	ns
SLC39A14	1.218487445	0.371516024	ns
SLC39A8	0.685903684	0.667706077	ns
SLC3A2	2.824507429	0.057224205	ns
SLC40A1	1.89955768	0.024881556	*
SLC7A11	0.078577926	0.004119928	**
SQSTM1	1.14534983	0.68696949	ns
STEAP3	1.223051531	0.618416811	ns
STIM1	1.331080518	0.507875198	ns

TF	0.67842826	0.623101179	ns
TFR1	0.561606141	0.253937898	ns
TFR2	0.553301429	0.410150011	ns
TP53	0.661163614	0.517095134	ns
TXNRD1	0.552808418	0.124791549	ns
USP7	0.70592808	0.177747585	ns
VDAC2	1.180957574	0.269214613	ns
VDAC3	0.76498527	0.009077216	**

Table S5

The results of PCR array in SMMC-7721

Target Name	SMMC-7721-DHA/SMMC-7721-NC	P value	
ACO1	1.359959902	0.624472217	ns
ACSL4	3.971413989	0.052266316	ns
AKR1B1	4.676905731	0.006600227	**
AKR1B10	0.146557011	0.055988532	ns
AKR1C1	7.843850953	0.0013332	**
ALDH1A1	0.073889193	0.000225747	***
ALOX15	0.12093607	0.013011384	*
ATF4	0.026671118	0.000477581	***
ATG5	2.103423008	0.053836399	ns
ATP5G3	5.639425715	0.005916041	**
BBC3	0.638927881	0.635524114	ns
BECN1	1.414437644	0.464336602	ns
BRAF	0.085169823	0.000305847	***
BRD4	0.328004267	0.067144151	ns
CA9	0.395550898	0.29803852	ns
CARS1	2.788752746	0.278528492	ns
CDO1	1.509237387	0.326265116	ns
CHAC1	0.455490547	0.171090653	ns
CISD1	0.670122976	0.54782327	ns
CISD2	1.035908915	0.964945284	ns
CP	0.123714239	0.008819467	**
CS	0.088694582	0.003873801	**
CYBB	0.635788975	0.620274663	ns
DMT1	34.77881401	0.016050016	*
DPP4	0.394894944	0.294628952	ns
ELAVL1	3.779484669	0.251341023	ns
EMC2	2.39210172	0.104198477	ns
EPRS	21.44022461	0.038633351	*
FTH1	1.239427426	0.535729069	ns
FTL	2.242991095	0.049619262	*
FTMT	0.556419658	0.288812266	ns
GCLC	3.922041744	0.235437452	ns
GCLM	2.75500034	0.109133611	ns
GLS2	0.697907739	0.3283133	ns
GOT1	3.535105922	0.011434842	*
GPX4	0.141652043	0.000922213	***
GSS	7.722598742	0.007315277	**
GSTA1	0.399491965	0.048867367	*
GSTP1	1.570658722	0.202292564	ns
HAMP	0.068805388	0.118556208	ns

HARS	2.006910266	0.025004368	*
HEPH	0.761516708	0.556678041	ns
HFE	0.802114343	0.693997826	ns
HMOX1	0.086538576	0.085019344	ns
HMOX2	1.923113592	0.429233319	ns
HRPT1	0.643104833	0.694517326	ns
HRAS	1.190118788	0.65533758	ns
HSF1	0.828348623	0.608141329	ns
HSPB1	1.337353446	0.327212104	ns
IREB2	0.181285126	0.001618746	**
KEAP1	0.622885188	0.322799713	ns
KRAS	0.725673799	0.387922288	ns
LOX	1.196114678	0.77726889	ns
LPCAT3	1.105718187	0.897776634	ns
MAP1LC3A	0.536829078	0.522227471	ns
MAP1LC3B	0.471143045	0.00845417	**
MAP1LC3C	0.432018774	0.164180379	ns
NCOA4	0.243460447	0.004446866	**
NFE2L2	0.531290257	0.077350524	ns
NOX1	1.393134231	0.715855341	ns
NOX3	0.134856801	0.334964032	ns
NOX4	0.420021292	0.299775448	ns
NQO1	5.586491624	0.001587259	**
NRAS	0.729378139	0.642011945	ns
PANX2	0.370846423	0.240604509	ns
PCBP1	1.422974889	0.607688298	ns
PCBP2	1.155704753	0.475520962	ns
PPARG	0.916354962	0.918596176	ns
PRDX6	1.04634191	0.76820497	ns
PRNP	1.562255076	0.138088482	ns
PTGES2	0.390847191	0.196891848	ns
RPL8	1.414698031	0.261755947	ns
SAT1	0.770839108	0.636639349	ns
SAT2	0.337372975	0.199662156	ns
SLC1A5	0.760114795	0.547794563	ns
SLC39A14	0.343788497	0.058570871	ns
SLC39A8	0.182026909	0.0090613	**
SLC3A2	0.195884061	0.178019018	ns
SLC40A1	0.407449012	0.287292181	ns
SLC7A11	0.350128914	0.019987353	*
SQSTM1	3.291135513	0.149845105	ns
STEAP3	1.162583088	0.911389715	ns
STIM1	2.731404511	0.316666896	ns

TF	0.10204083	0.071472769	ns
TFR1	0.742807481	0.598199215	ns
TFR2	0.17484198	0.015494982	*
TP53	0.107812231	0.001272787	**
TXNRD1	1.831228457	0.219922185	ns
USP7	0.57970841	0.477702826	ns
VDAC2	0.843468791	0.604115592	ns
VDAC3	0.662145659	0.501575269	ns

Table S6

Clinical characteristics of HCC patients.

Characteristic	Low expression of ATF4	High expression of ATF4	*p*
n	187	187	
T stage, n (%)			0.004
T1	108 (29.1%)	75 (20.2%)	
T2	39 (10.5%)	56 (15.1%)	
T3	31 (8.4%)	49 (13.2%)	
T4	7 (1.9%)	6 (1.6%)	
N stage, n (%)			0.622
N0	127 (49.2%)	127 (49.2%)	
N1	1 (0.4%)	3 (1.2%)	
M stage, n (%)			0.369
M0	132 (48.5%)	136 (50%)	
M1	3 (1.1%)	1 (0.4%)	
Pathologic stage, n (%)			0.004
Stage I	101 (28.9%)	72 (20.6%)	
Stage II	37 (10.6%)	50 (14.3%)	
Stage III	33 (9.4%)	52 (14.9%)	
Stage IV	4 (1.1%)	1 (0.3%)	
Age, n (%)			0.133
<=60	81 (21.7%)	96 (25.7%)	
>60	106 (28.4%)	90 (24.1%)	
Gender, n (%)			0.122
Female	53 (14.2%)	68 (18.2%)	
Male	134 (35.8%)	119 (31.8%)	
Histologic grade, n (%)			0.142
G1	30 (8.1%)	25 (6.8%)	
G2	97 (26.3%)	81 (22%)	
G3	52 (14.1%)	72 (19.5%)	
G4	5 (1.4%)	7 (1.9%)	
AFP (ng/ml), n (%)			< 0.001
<=400	128 (45.7%)	87 (31.1%)	
>400	19 (6.8%)	46 (16.4%)	
Vascular invasion, n (%)			0.010
No	120 (37.7%)	88 (27.7%)	
Yes	46 (14.5%)	64 (20.1%)	

Abbreviations: HCC, hepatocellular carcinoma; ATF4, activated transcription factor 4.

Table S7

The ATF4 potential binding sites in slc7a11, predicted by the Jaspar database (Jaspar.genereg.net)

Matrix ID	Name	Score	Relative score	Sequence ID	Start	End	Strand	Predicted sequence
MA0833.2	MA0833.2.ATF4	13.095031	0.9067711927402915	FP007408	1922	1935	-	tgatgatgcaaatt
MA0833.2	MA0833.2.ATF4	11.500193	0.8852120829967531	FP007408	1903	1916	+	ggctgatgcaaacc
MA0833.1	MA0833.1.ATF4	11.210324	0.87747116852988	FP007408	1903	1915	+	ggctgatgcaaac
MA0833.2	MA0833.2.ATF4	10.071239	0.865895415425158	FP007408	1548	1561	+	aaattatgaaattc
MA0833.1	MA0833.1.ATF4	9.789632	0.8575957820923111	FP007408	1923	1935	-	tgatgatgcaaat
MA0833.2	MA0833.2.ATF4	8.148878	0.8399088286205307	FP007408	1667	1680	+	aaatgaagtaacta
MA0833.2	MA0833.2.ATF4	7.691121	0.8337208443645527	FP007408	456	469	-	tcctcatgcaatcc
MA0833.1	MA0833.1.ATF4	7.5258994	0.825926326047608	FP007408	457	469	-	tcctcatgcaatc
MA0833.1	MA0833.1.ATF4	7.5194397	0.8258359551215482	FP007408	1501	1513	-	ttataaggcaata
MA0833.2	MA0833.2.ATF4	7.089798	0.8255921252439103	FP007408	294	307	-	ctctgaagaaatat
