# Supplementary material for: Dihydroartemisinin induces ferroptosis of hepatocellular carcinoma via inhibiting ATF4‐xCT pathway
Source: J Cell Mol Med. 2024 Apr 23;28(8):e18335. doi: 10.1111/jcmm.18335 (PMC11037408; doi:10.1111/jcmm.18335)
Supplement: Supplementary file 3 — Figures S1–S5. [file JCMM-28-e18335-s003.pdf]

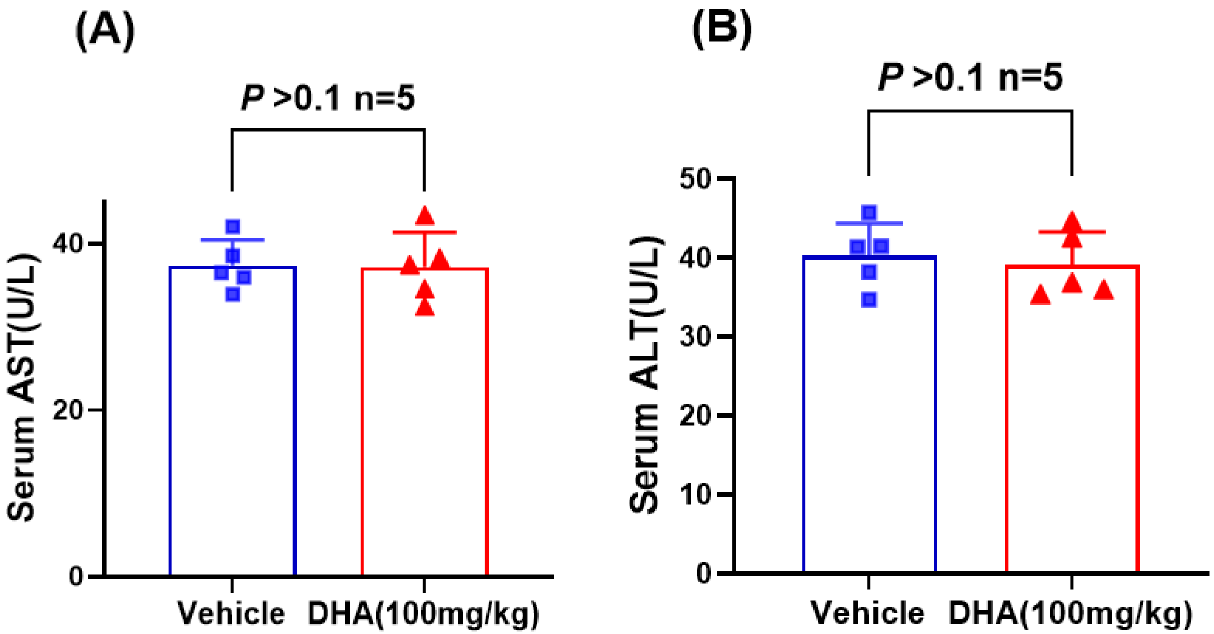

Fig. S1 DHA has no hepatotoxicity in mice. (A) and (B): the levels of serum ALT and AST are presented as mean ± SD. (n=5; *P*>0.1).

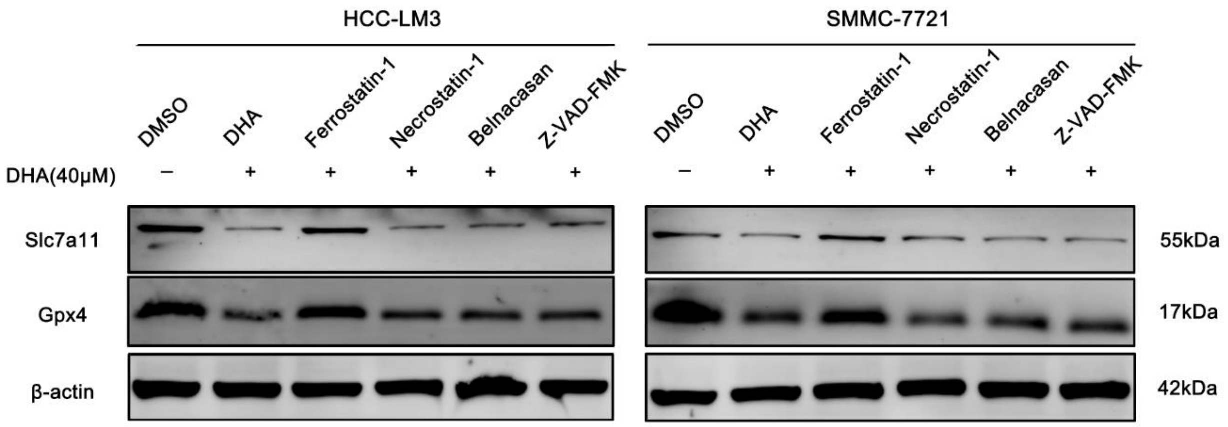

Fig. S2 Western blotting analysis of SLC7A11 and GPX4 in cell lysates of HCC-LM3 and SMMC-7721 treated with DMSO, DHA, DHA + ferrostatin-1, DHA + Necrostatin-1, DHA + Belnacasan or DHA + Z-VAD-FMK.

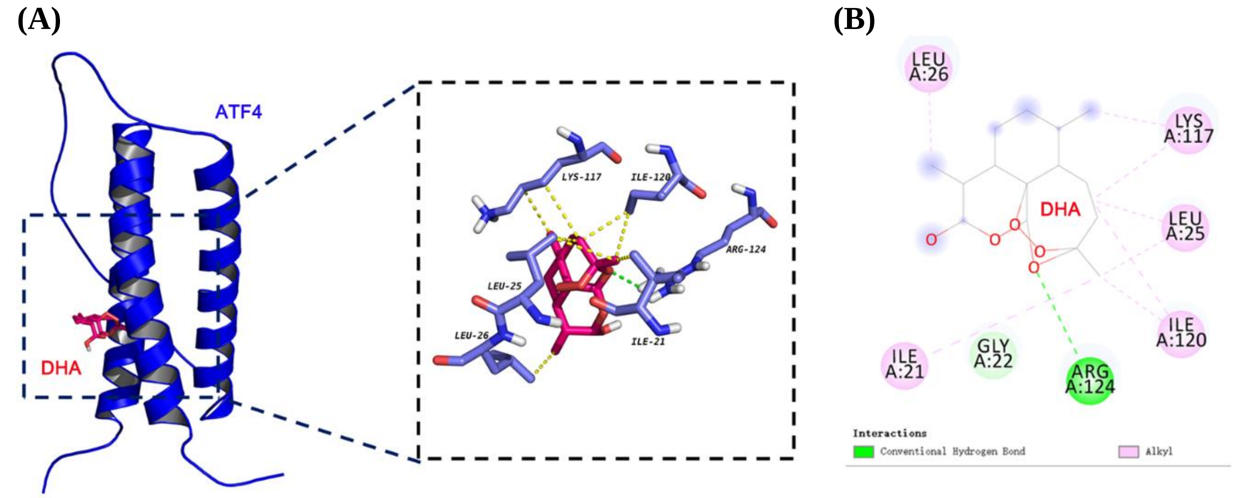

Fig. S3: Molecular docking analysis of DHA and ATF4. (A) Crystal structure of DHA bound to ATF4. (B) Plane model diagram of interaction force between DHA and ATF4.

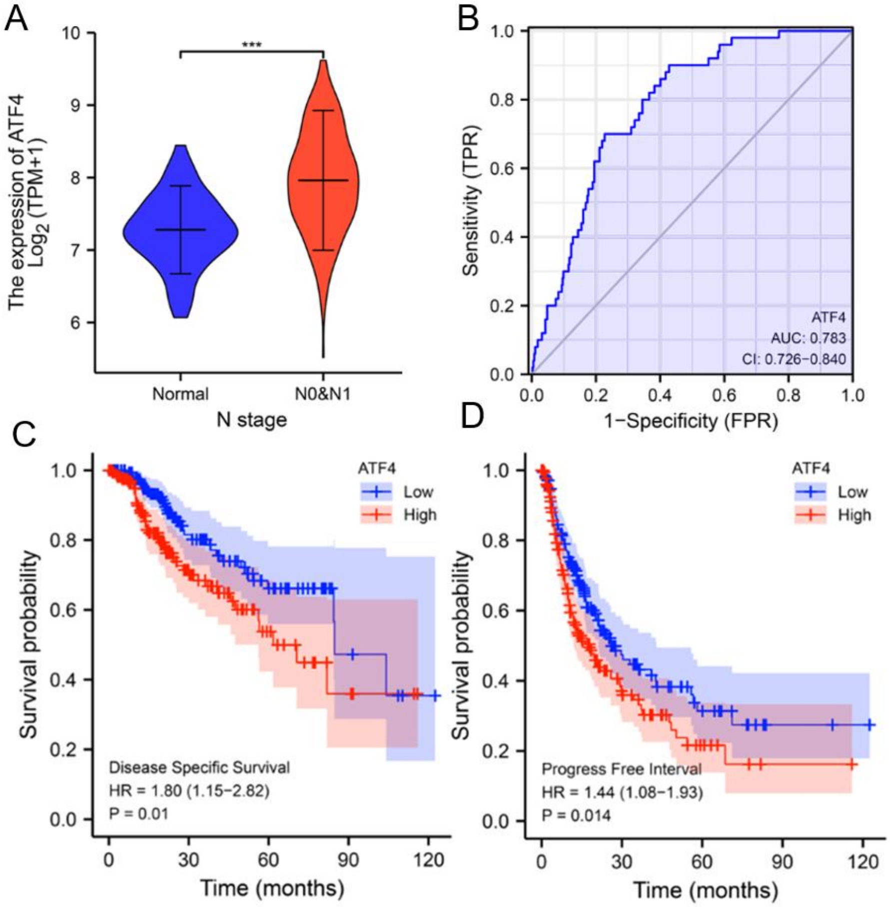

Fig. S4 The high expression of ATF4 is associated with poor clinicopathological features of HCC. (A)Relationship between ATF4 expression and N-stage in HCC. (B) ROC curve of ATF4 distinguishing HCC tissues. (C-D) Kaplan-Meier curves for disease-specific survival and progress free interval. Data represent means ± SD, *p < 0.05, **p < 0.01 and ***p < 0.001.

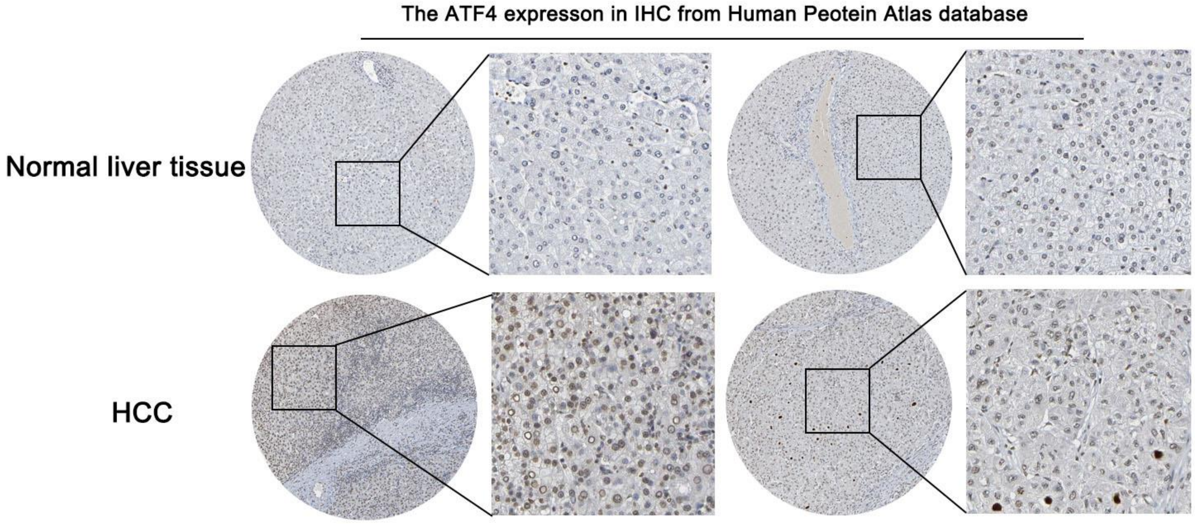

Fig.S5: Immunohistochemical results of ATF4 in HCC tissue and normal liver tissue from The Human Protein Atlas Database (https://www.proteinatlas.org).
